# The state and significant drivers of health systems efficiency in Africa: A systematic review and meta-analysis

**DOI:** 10.7189/jogh.13.04131

**Published:** 2023-11-09

**Authors:** Juliet Nabyonga-Orem, Christmals Christmal, Kingsley F Addai, Kasonde Mwinga, Kizito Aidam, Gilbert Nachinab, Sylivia Namuli, James A Asamani

**Affiliations:** 1 World Health Organization (WHO) Africa Regional Office, Office of the Regional Director, Brazzaville, Congo; 2 Centre for Health Professions Education, Faculty of Health Sciences, North-West University, Potchefstroom, South Africa; 3 World Health Organization (WHO) Ghana Country Office, Universal Health Coverage Life Course Cluster, Accra, Ghana; 4 World Health Organization (WHO) Africa Regional Office, Universal Health Coverage Life Course Cluster, Brazzaville Congo; 5 Appalachian School of Law, Virginia, USA; 6 University of Development Studies, Tamale, Ghana

## Abstract

**Background:**

Low-and-middle-income countries, especially in Africa, lack the capacity to adequately invest in health systems to attain universal health coverage (UHC). As such, countries must improve efficiency and provide more services within the available resources. This systematic review synthesised evidence on the efficiency of health systems in the African region and its drivers.

**Methods:**

We conducted a systematic literature review guided by the Preferred Reporting Items for Systematic Reviews and Meta-Analyses (PRISMA) 2020 statement. Related studies were grouped and meta-analysed, while others were descriptively analysed. We employed a qualitative content synthesis for synthesising the drivers of efficiency.

**Results:**

Overall, 39 studies met a predetermined inclusion criterion and were included from a possible 4 609 records retrieved through a rigorous search and selection process. Using a random effects restricted maximum likelihood method, the pooled efficiency score for the Africa region was estimated to be 0.77, implying that on the flip side, health system inefficiency across countries in the African region was approximately 23%. Across 22 studies that used data envelopment analysis to examine efficiency at the level of health facilities and sub-national entities, the efficiency level was 0.67. Facility-level studies tended to estimate low levels of efficiency compared to health system-level studies. Across the 39 studies, 21 significant drivers of inefficiency were reported, including population density of the catchment area, governance, health facility ownership, health facility staff density, national economic status, type of health facility, education index, hospital size and bed occupancy rate.

**Conclusion:**

With approximately 23% of the inefficiency of health systems in Africa, improving efficiency alone will yield an average of 34% improvement in resource availability, assuming all countries are performing similarly to the frontier countries. However, with the low level of health expenditure per capita in Africa, the efficiency gains alone will be insufficient to meet the minimum funding requirement for UHC.

**Registration:**

PROSPERO: CRD42022318122.

Efficiency measures the extent to which resources produce their desired results [1]. Efficiency exists in two major types, technical efficiency and allocative efficiency. Allocative efficiency measures how various resource inputs interact to give off outputs [2]. On the other hand, technical efficiency involves determining how maximum output could be achieved out of the least amount of investment [2]. Due to methodological complexities, assessing the efficiency of healthcare systems is challenging [3]. In recent decades, data envelopment analysis (DEA) has been employed in several countries to examine the efficiency of health systems [2-7]. As such, DEA seems to be a reliable and appropriate approach in quantitative assessment of the efficiency of healthcare institutions and health systems [3].

DEA uses selected healthcare system variables to compute its efficiency score(s) through linear programming and frontier estimation techniques [3]. The DEA model is either input or output-oriented [3]. An output-oriented DEA model is channelled towards maximising the outputs obtained while inputs are kept constant. On the contrary, input-oriented DEA models focus on minimising the inputs used for attaining a certain level of selected outputs. The type of input and output variables used in DEA have varied widely across studies, often determined by data availability and the objectives of a specific study [3-5,8]. In broad terms, inputs used in DEA models often include human resources, medicines and health technology, level of health expenditure, and physical structure. Output variables usually include the number of consultations/visits, inpatient care indicators and health status indicators. Specifically, consultation visits consist of data on outpatient, inpatient, and special care visits [5]. Others focus on maternal and child health services output such as antenatal care visits, delivery, immunisation, postnatal visits, and family planning visits [4,5] or allied health services, including health education sessions, tests and observations, and community engagement activities [5]. Typical impact indicators used as output variables are mortality rate (all sub-types), life expectancy, and health-adjusted life expectancy [3].

Inefficiency in healthcare systems is alarming in Sub-Saharan Africa (SSA) health systems. For example, one systematic review showed that the technical efficiency of healthcare systems in Eastern Africa ranged from 41% to 69%, while the technical and allocative efficiency of health systems in West Africa was just 40% or less [5]. Similarly, in Southern Africa, the allocative efficiency of healthcare systems was 40% [5]. Technical and allocative efficiency of some health centres in Ghana were found to be very poor, 22% and 18% respectively [4].

Various drivers of efficiency have been documented in SSA. Newer health facilities and facilities that were given some form of incentives were observed to be more efficient than older facilities and facilities that were not given any incentives [4]. In resource-constrained settings, healthcare systems must function efficiently to maximise the outputs from the limited resources available [9]. As such, reviewing available literature on the efficiency of healthcare systems in SSA and its drivers is sacrosanct. This will equip policymakers with the necessary scientific evidence that is required in making evidence-informed policies towards enhancing the efficiency of healthcare systems.

## METHODS

We conducted a systematic literature review guided by the Preferred Reporting Items for Systematic Reviews and Meta-Analyses (PRISMA) 2020 statement [10]. Moreover, we employed the sample, phenomenon of interest, design, evaluation, and research type (SPIDER) mnemonics in the search, inclusion, and exclusion of studies [11].

### Review question

To shape the review question, a scoping search showed a potentially large volume of literature on the topic. Based on the preliminary findings from the scoping search, the review questions explored in this study were: what is the level of efficiency of health systems in the World Health Organization (WHO)-Africa region, and what are the significant drivers of health system (in)efficiency in the WHO-Africa region? The following criteria were set: a) sample: national and subnational health systems in WHO-Africa region, b) phenomenon of interest: efficiency of health systems, c) design: peer-reviewed and grey literature of any study design, d) evaluation: level of efficiency and significant drivers of efficiency, and e) research type: quantitative and qualitative studies published from 2011 to 2021.

### Inclusion and exclusion criteria

We included all peer-reviewed and grey literature conducted using any research design on the efficiency and its drivers in the WHO-Africa member countries’ health systems. Further, we included studies published in English from January 2011 to July 2021.

### Search strategy

We searched PubMed, Scopus, ProQuest and EbscoHost (Medline and CINAHL) databases using a Boolean combination of the keywords efficiency, drivers, health system and Africa. However, including Africa made the search complex; therefore, Africa and its alternatives were eliminated and instead used as the exclusion criterion. Depending on the database, the keywords were combined or replaced with their alternatives in search strings.

### Search string

The master search string was (*Efficiency OR performance) AND (health system* OR hospital* OR health care service* OR *clinic). Other derivative forms of the search string used were (inefficiency OR performance) AND (health systems OR hospitals OR health care services OR clinic), and (efficiency OR performance) AND (health systems OR hospitals OR health care services OR clinic) AND (Countries in the WHO Africa region). For details see **Table 1**.

**Table 1:** Search terms

Keywords	Alternatives words used
**Efficiency**	Performance
**Health system**	Hospital, health centre, healthcare services clinic
**Africa**	Countries in the WHO Africa region*, West Africa, East Africa, Southern Africa

WHO – World Health Organization

*Algeria, Angola, Benin, Botswana, Burkina Faso, Burundi, Cabo Verde, Cameroon, Central African Republic, Chad, Comoros, Congo, Cote d’Ivoire, Democratic Republic of Congo, Equatorial Guinea, Eritrea, Eswatini, Ethiopia, Gabon, Gambia, Ghana, Guinea, Guinea Bissau, Kenya, Lesotho, Liberia, Madagascar, Malawi, Mali, Mauritania, Mauritius, Mozambique, Namibia, Niger Nigeria, Rwanda, Sao Tome and Principe, Senegal, Seychelles, Sierra Leone, South Africa, South Sudan, Togo, Uganda, United Republic of Tanzania, Zambia, Zimbabwe.

Forward and backward citation searches were conducted on relevant studies identified [12]. We contacted key authors and practitioners within the WHO-Africa region to recommend studies that we had difficulties retrieving or might have missed in the computerised search. CC, KA, and NT independently reviewed the studies for inclusion. Studies that were preliminarily included through the consensus of the reviewers were critically appraised before final inclusion. JNO, CC, JA, KA and NTG conducted an in-depth review of selected papers and data mining.

### Studies included

**Figure 1:**
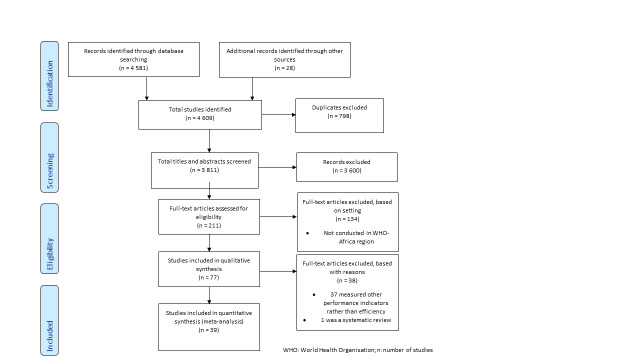
The search and the inclusion process for studies. WHO – World Health Organization.

The computerised search yielded 4 581 studies, but through a process evaluation and critical appraisal, only 39 studies met at least 75% of the critical appraisal criteria and were therefore included for data extraction and synthesis (**Figure 1**). Furthermore, we evaluated 39 studies for their suitability for meta-analysis, i.e. used DEA methodology, reported results for multiple countries, and used health systems as a unit of analysis. Five studies [6,13-16] met these criteria and were included in the meta-analysis. For this study, we adapted the critical appraisal tool used by Babalola et al. [5]. The appraisal tool and the results of the critical appraisal process are presented in the Table S1 in the **Online Supplementary Document****.**

### Data extraction

We extracted the relevant content of the included papers onto the priori data matrix (Table S2 in the **Online Supplementary Document**). Among these are author, year, methodology, types and scope, efficiency level and significant drivers.

### Data analysis

Data analysis was conducted in three parts. A meta-analysis of system-level studies that used DEA, a descriptive synthesis of the level of efficiency of subnational/institutional efficiency studies and a narrative synthesis [12,17] of qualitative results of the drivers of efficiency. We analysed the number, methodology and sampling of the quantitative studies to select studies suitable for meta-analysis [18]. We used the Stata Statistical Software (Release 16, StataCorp LLC, College Station, Texas) to conduct the meta-analysis of the health system-level efficiency scores, and the results were presented in summary statistics and forest plot. To account for the underlying heterogeneity in the inputs and outputs measures of efficiency across different studies, the random-effects model of meta-analysis was employed to overcome the limitation [19,20]. The levels of heterogeneity in the results were statistically estimated, and as far as possible, sensitivity analysis was used to examine the robustness of the findings. Facility-level or subnational-level studies that were not feasible to pool together in the meta-analysis were presented in the form of descriptive summary statistics and descriptively compared with the pooled efficiency score from the meta-analysis of health system-level efficiency. The nature of the data did not allow for further inferential analysis.

## RESULTS

### Descriptive analysis of the included studies

#### The country setting of included studies

Out of the 39 studies included, 27 were country-specific, including Burkina Faso [21], Eritrea [22], Ethiopia [23-27], Gambia [28] Ghana [29-32], Kenya [33,34], Mauritius [35], Mozambique [36], Nigeria [37,38], Rwanda [39], Sierra Leone [40], South Africa [5,41,42], Togo [43], Uganda [44,45] and Zambia [46]. The rest (12 studies) were multicountry studies [6,13-16,47-53] covering 46 out of the 47 countries in the WHO-Africa region. South Sudan was the only country from which no efficiency studies were found.

The highest number of studies were recorded in the following countries (n = 9 for each country) Ethiopia, Ghana, Kenya, South Africa, and Uganda. Regarding country-specific studies, Ethiopia had the highest studies (n = 5), followed by Ghana (n = 4), and South Africa (n = 3). With respect to multicountry studies, Kenya, Uganda, Rwanda, Botswana, and Guinea had the highest studies (n = 7 for each country).

#### Scope and types of efficiency analysis conducted

All the studies included were on technical efficiency and its drivers. 27 studies used health facility or sub-national level as a unit of analysis, including 22 DEA and five Stochastic Frontier Analysis (SFA) studies. Across these 27 studies, data from 997 hospitals, 112 health centres, and 125 health posts were used for the analysis. The other 12 were multicountry in nature and used the health system as the unit of analysis. Of these, nine used DEA, two SFA and one combined the two approaches using the ensemble method.

#### Input variables used for efficiency analysis

We synthesised the inputs and outputs under system-level studies and health facility/sub-national level studies. Out of the 12 system-level efficiency studies included, expenditure (n = 10) was the most used input variable [6,13-16,47,49,50,52,53], followed by total health workforce (n = 6) [6,13,47,48,51,52], and capacity (n = 3) [6,48,51]. Other input variables included unemployment rate [6,50], immunisation rate, Gini coefficient [50,53], educational index [49,50], age group of participants [14,50], food production index, inflation rate, purchasing power parity, urbanisation [49], external resources [14], and use of sanitation services [50].

Regarding health facility/sub-national level efficiency studies, workforce (n = 23), bed capacity (n = 15) and expenditure (n = 14) are the main inputs (Table S3 in the **Online Supplementary Document**). Other inputs included capital cost, number of admissions, number of specific services provided, equipment index, hospital space in square meters, depreciation, vaccines, time spent on service provided, medicines, insurance coverage and access to water.

#### Output variables used for efficiency analysis

Regarding system-level studies, life expectancy (n = 8), and mortality rate (n = 7) were the major output variables. Other output variables included outpatient visits (n = 3), admissions (n = 2), infection rate (n = 2), immunisation rate (n = 2), antenatal coverage (n = 1), Antiretroviral therapy visits (n = 1), number of deliveries (n = 1), expenditure (n = 1), access to healthcare (n = 1), skilled labour attendance (n = 1) and treatment success rate (n = 1) (Table S3 in the **Online Supplementary Document**). For health facility/subnational efficiency studies, outpatient visits (n = 14), inpatient admissions (n = 11), number of deliveries (n = 7), mortality rate (n = 4), immunisation rate (n = 4), antenatal coverage (n = 40), and number of reproductive health visits (n = 4) were the major output variables measured. Other variables included laboratory investigations conducted (n = 2), surgical operations (n = 2), number of x-rays done (n = 2), ART visits (n = 2), postnatal visits (n = 2), skilled birth attendance (n = 1), treatment success rate (n = 1), quality of service (n = 1), postnatal coverage (n = 1), and competition with other health facilities(n = 1).

### Exploring the state of health systems efficiency in Africa from published literature

We present the efficiency of the health systems in two ways: the meta-analysis and a descriptive synthesis of the papers not included in the meta-analysis.

#### Meta-analysis of health system-level multicountry studies that used data envelopment analysis (DEA)

A subset of five out of the 39 included studies (13%) used reasonably similar DEA methodology, albeit with variations in inputs and output variables and were considered suitable for meta-analysis. Of these, three [13,14,16] undertook global analysis and included a large number of African countries. Only one study [6] focused on only African countries. For the three studies that analysed the efficiency of health systems globally, the scores for African countries were theoretically benchmarked against frontier countries outside of Africa. The average efficiency scores for the African region across these four studies ranged from 0.619 to 0.928. Another study included 43 African countries amongst 172 countries globally [15].

**Figure 2:**
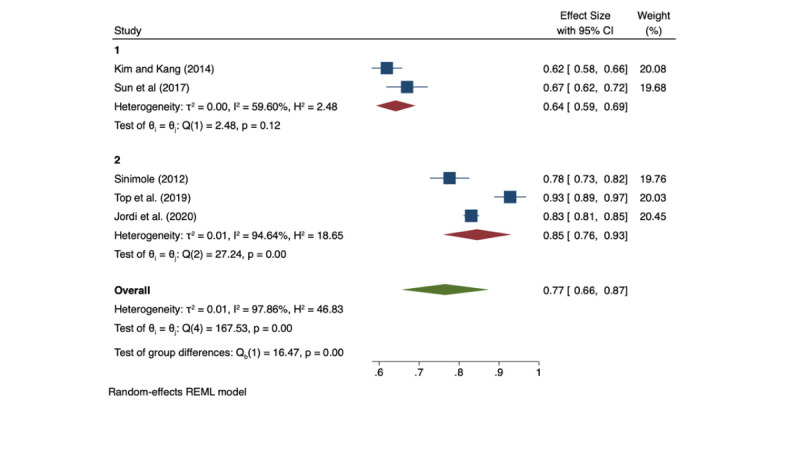
Forest plot on efficiency of health systems in Africa (multicountry data envelopment analysis studies). CI – confidence interval, REML – restricted maximum likelihood.

Using the random effects restricted maximum likelihood (REML) method, the pooled efficiency score for the African region was estimated to be 0.77 (95% confidence interval (CI) = 0.66-0.83). On the flip side, health system inefficiencies across countries in the African region were approximately 23%. However, when the 95% CI is taken into account, the level of inefficiency could be as much as 34% or as low as 17%. This wide CI in the level of inefficiency reflects the vast heterogeneity in methodologies, input and output variables as well as data, and timeframes of the studies included in the meta-analysis (*I^2^* = 97.86%). **Figure 2** shows the forest plot of the pooled estimate of health system efficiency in Africa with the inherent levels of uncertainty and heterogeneity amongst the studies included. A detailed summary of the studies that estimated the health systems level of technical efficiency across countries is shown in **Table 2**, with the regional average ranging from 0.55 to 0.96.

**Table 2:** Summary of health systems technical efficiency scores from multicountry Data Envelopment Analysis (DEA) studies

	Technical efficiency scores (VRS) by paper				Summary		
**Countries**	**Jordi et al. (2020)**	**Top et al. (2019)**	**Sinimole (2012)**	**Kim and Kang (2014)**	**Number of studies included**	**Low estimate**	**High estimate**
Algeria	0.97	1.00		0.84	3	0.84	1.00
Angola	0.77			0.54	2	0.54	0.77
Benin	0.80	1.00		0.70	3	0.70	1.00
Botswana	0.97	0.64		0.65	3	0.64	0.97
Burkina Faso	0.73	0.99		0.80	3	0.73	0.99
Burundi	0.97			0.33	2	0.33	0.97
Cameroon	0.71	0.88		0.52	3	0.52	0.88
Cabo Verde	0.94	1.00		1.00	3	0.94	1.00
Central African Republic	1.00			0.32	2	0.32	1.00
Chad	0.65	1.00		0.71	3	0.65	1.00
Comoros	0.82		1.00	1.00	3	0.82	1.00
Congo, Rep	0.84		0.84	0.68	3	0.68	0.84
Côte d'Ivoire	0.70			0.87	2	0.70	0.87
Democratic Republic of the Congo	1.00			0.20	2	0.20	1.00
Equatorial Guinea	0.81			1.00	2	0.81	1.00
Eritrea			1.00	1.00	2	1.00	1.00
Eswatini	0.92			0.44	2	0.44	0.92
Ethiopia	0.85	1.00		0.92	3	0.85	1.00
Gabon	0.82	1.00		0.91	3	0.82	1.00
Gambia	0.87	0.95	1.00	0.49	4	0.49	1.00
Ghana	0.77	0.94		0.40	3	0.40	0.94
Guinea	0.78	1.00	0.87	1.00	4	0.78	1.00
Guinea-Bissau	0.71	0.76	1.00	0.83	4	0.71	1.00
Kenya	0.94	0.90		0.34	3	0.34	0.94
Lesotho	0.86			0.49	2	0.49	0.86
Liberia	0.71	1.00	1.00	0.42	4	0.42	1.00
Madagascar		1.00		0.31	2	0.31	1.00
Malawi	0.94	1.00		0.35	3	0.35	1.00
Mali	0.56	1.00		0.66	3	0.56	1.00
Mauritania	0.59	1.00		0.78	3	0.59	1.00
Mauritius			0.14	0.78	2	0.14	0.78
Mozambique	1.00	0.86		0.39	3	0.39	1.00
Namibia	0.95			0.64	2	0.64	0.95
Niger	0.74			1.00	2	0.74	1.00
Nigeria		1.00		0.68	2	0.68	1.00
Rwanda	0.89	1.00		0.34	3	0.34	1.00
Sao Tome and Principe	0.93		0.68	0.32	3	0.32	0.93
Senegal	0.76	1.00		0.33	3	0.33	1.00
Seychelles	1.00		0.14	0.58	3	0.14	1.00
Sierra Leone	0.54	1.00	0.87	0.74	4	0.54	1.00
South Africa	0.96	0.53		0.84	3	0.53	0.96
United Republic of Tanzania	0.82	1.00		0.27	3	0.27	1.00
Togo	0.78	0.91		0.76	3	0.76	0.91
Uganda	0.75	0.91		0.30	3	0.30	0.91
Zambia	0.88	0.74		0.43	3	0.43	0.88
Zimbabwe	0.88	0.85			2	0.85	0.88
Regional Average	0.83	0.93	0.78	0.62	3	0.55	0.96

#### Sub-group and sensitivity analysis

To assess the impact of study characteristics on the pooled effect of the meta-analysis, we conveniently grouped the studies into group one that comprised two studies that had low efficiency scores (lower than 0.70) and group two comprising three studies that had high efficiency scores (at least 0.70).

**Figure 3:**
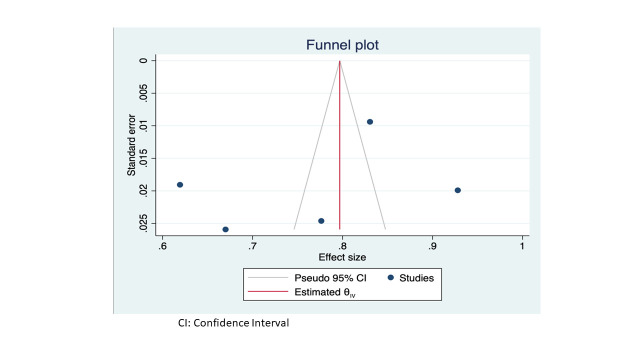
Funnel plot. CI – confidence interval.

The analysis showed that studies with low scores had a pooled efficiency score of 64% (95% CI = 59-69), which is 13% lower than the overall pooled efficiency score of 77%. Although this difference was statistically insignificant (*P* value (*P*) = 0.57) when tested with Egger’s test for small-study effects, the degree of variation should still be considered important. On the other hand, group two studies yielded a pooled score of 85% (95% CI = 81-85), which was significantly different from the group one results by 21% (*P* < 0.05). However, the CI of the pooled group two studies overlaps with the CI of the overall pooled efficiency score (76%-87%), suggesting an insignificant difference (**Figure 3**). On the other hand, group two studies yielded a pooled score of 85% (95% CI = 81-85), which was significantly different from the group one results by 19% (*P* < 0.05). However, the CI of the group two studies pooled overlaps with the CI of the overall pooled efficiency score (76- 87%), suggesting an insignificant difference (**Figure 3**).

22 studies representing 56.40% of all the studies included in the systematic review conducted health facility-level or sub-national level efficiency analyses in 12 countries using the DEA method. The data extracted from these studies was not suitable for meta-analysis, hence is descriptively presented in **Table 3**. Across these 22 studies from 12 countries, the level of efficiency varied widely from 0.42 in the KwaZulu Natal province of South Africa [54] to 0.90 in Eritrea [22]. The average efficiency score across these studies was 0.67 (median efficiency score = 0.65) as compared to the Africa pooled efficiency score of 0.77 (95% CI = 0.66-0.87) from the meta-analysed multicountry studies focusing on broad health system indicators beyond health facility outputs. Since the central tendency (average and median) of the efficiency scores from the health facility level studies fall within the 95% CI of the pooled overall efficiency score from the meta-analysis of health system-level multicountry studies, the difference of 10% will unlikely be statistically significant. As shown in **Table 4**, across the five facility-level or sub-national level SFA studies, the average efficiency score was 0.72 (median score = 0.73) but ranged widely from 0.51 in Ghana to 0.83 in Mauritania, which crosses the 95% CI = 0.66-0.87 estimated from the pooled overall estimate in the meta-analysis.

**Table 3:** Descriptive summary of efficiency scores from health facility-level or sub-national level Data Envelopment Analysis (DEA) studies

Study (year)	Country	Method used	Average efficiency score
Achoki et al. (2017) [46]	Zambia	DEA	0.62
Jehu-Appiah et al. (2014) [29]	Ghana	DEA	0.61
Yitbarek et al. (2019) [23]	Ethiopia	DEA	0.59
Zeng et al. (2014) [39]	Rwanda	DEA	0.78
Babalola and Moodley (2020) [54]	South Africa	DEA	0.42
Ali et al. (2017) [24]	Ethiopia	DEA	0.78
Ichoku et al. (2011) [37]	Nigeria	DEA	0.59
Ngobeni et al. (2020) [42]	South Africa	DEA	0.64
Alhassan et al. (2015) [32]	Ghana	DEA	0.65
Ayiko et al., (2020) [44]	Uganda	DEA	0.49
Yitbarek et al. (2019) [25]	Ethiopia	DEA	0.85
Kinyanjui et al. (2015) [33]	Kenya	DEA	0.59
Kirigia and Asbu (2013) [22]	Eritrea	DEA	0.90
Marschall and Flessa (2011) [21]	Burkina Faso	DEA	0.62
Aduda etl al. (2015) [34]	Kenya	DEA	0.76
Amare et al. (2020) [26]	Ethiopia	DEA	0.74
Mujasi et al. (2016) [45]	Uganda	DEA	0.79
Zeng et al. (2014) [39]	Rwanda	DEA	0.72
Bobo et al. (2018) [27]	Ethiopia	DEA	0.77
Kirigia et al. (2011) [40]	Sierra Leone	DEA	0.43
Jarue et al. (2015) [28]	Gambia	DEA	0.65
**Additional explanation**			
Mean			0.67
Median			0.65
Standard deviation			0.13
Minimum			0.42
Maximum			0.90

DEA – data envelopment analysis

**Table 4:** Descriptive summary of efficiency scores from health facility-level or sub-national level Stochastic Frontier Analysis studies

Study (year)	Country	Analytical method used	Average efficiency score
Nundoochan (2020) [35]	Mauritania	SFA	0.83
Novignon and Nonvignon (2017) [30]	Ghana	SFA	0.51
Anselmi et al. (2018) [36]	Mozambique	SFA	0.73
Ichoku et al. (2014) [38]	Nigeria	SFA	0.71
Kinfu (2015) [41]	South Africa	SFA	0.81
**Additional explanation**			
Mean			0.72
Median			0.73
Standard deviation			0.13
Minimum			0.51
Maximum			0.83

SFA – stochastic frontier analysis

### Comparison between health facility level efficiency estimates and multicountry estimates

When the studies that used health facilities as the unit of analysis were compared with the multicountry studies that used the entire health system as the unit of analysis, a systematic difference was observed. Across the 12 countries, the variation between the facility-level and the system-level estimates ranges from 0.10 in Eritrea to 0.35 in Sierra Leone, with an average of 0.19. In all the countries, the health facility-level studies tended to produce lower efficiency scores compared to multicountry health system-level studies (**Table 5**).

**Table 5:** Comparison between health facility-level efficiency and health system-level efficiency estimates across 12 countries (studies that used data envelopment analysis)

Country	Number of health facility-level studies	Number of health system-level studies	Average score from health facility-level studies (a)	Average score from health system-level studies (b)	Variance (c = a-b)
Zambia	1	3	0.62	0.77	-0.15
Ghana	2	3	0.63	0.81	-0.18
Ethiopia	5	3	0.75	0.93	-0.18
Rwanda	2	3	0.75	0.88	-0.13
South Africa	3	3	0.62	0.75	-0.12
Nigeria	2	2	0.65	0.94	-0.29
Uganda	2	3	0.64	0.77	-0.13
Kenya	2	3	0.68	0.86	-0.18
Eritrea	1	2	0.90	1.00	-0.10
Burkina Faso	1	3	0.62	0.86	-0.24
Sierra Leone	1	4	0.43	0.78	-0.35
Gambia	1	4	0.65	0.88	-0.23
**Additional explanation**					
Mean			0.66	0.85	-0.19
Median			0.65	0.86	-0.18
Minimum			0.43	0.75	-0.35
Maximum			0.90	1.00	-0.10

#### Synthesis of the drivers of (in)efficiency across the studies

The studies reported many drivers of health system efficiency, depending on the specific focus of the efficiency study. The drivers were synthesised according to the classification of studies reporting them – system-wide or health facility-level studies.

#### Population density of catchment area

Population density of the catchment area was the most frequently reported driver of the efficiency of health systems. Obure et al. [47] and See and Yen [48] found that lower population density of the location of the facility reduced efficiency with β = -0.062 to -0.0187, which is the average change in efficiency given a unit change in population density, other factors being equal. In other words, they found that higher population density of the catchment area of the health facility and efficiency were positively associated. However, facility/sub-national efficiency studies reported varying relationships between the population density of the catchment area and efficiency, with coefficients ranging from β = -0.0524 to 0.0000078. Four studies [22,23,25,27] reported that the bigger the size of the catchment area or an increase in the population density of the catchment area, the better the efficiency. On the other hand, Amare et al. [26] reported that a 100 000 increment in the catchment population of the facility decreases efficiency (β = -0.0524). With the frequency of the evidence supporting the positive relation, the authors concluded that increasing the population density increases the efficiency of the health system.

#### Governance

Good governance was positively correlated with high-efficiency scores of health systems [16,48,53]. For instance, Jordi et al. [16] and Ibrahim et al. [53] both reported that effective governance and efficiency are positively correlated (*r* = 0.027, β = 0.2). Affirmatively, See and Yen [48] found governance effectiveness negatively correlated with inefficiency (β = -0.0399). In comparison with other drivers or factors, Ibrahim and colleagues [53] discovered that governance measures had a stronger impact on the efficiency of health systems than expenditure on public health, implying that the management of resources is to be prioritised over the injection of resources. At the facility/sub-national level, Alhassan et al. [55], however, reported that governance measures on environmental safety in the health facility and efficiency were negatively correlated (*r* = -0.2764).

#### Type of health facility ownership

There were varying reports on the association between the type of health facility and the efficiency of health systems. One system-level study [47] and three facility-level studies [24,32,39] reported that public and quasi-government ownership were positively correlated with efficiency. Specific to private health facilities, Ichoku et al. [38] found that privately owned hospitals were more efficient than public hospitals. This contradicted the findings of Jehu-Appiah et al. [29], stating that privately owned hospitals were less efficient.

#### Hospital staff density

Among system-level studies, Obure et al. [47] reported that the proportion of clinical staff to overall staff is negatively correlated with inefficiency (*r* = -0.431). Top et al. [6] confirmed this finding, reporting that a higher number of nurses per 1 000 people increases efficiency (*r* = 0.125). Among facility-level studies, Atake [43] and Ali et al. [24] reported that low medical staff density leads to higher inefficiency (*r* = - 0.765, *r* = -26.65).

#### National economic status

Two health system-level studies [15,53] reported the relationship between national economic outlook and efficiency. In an economic model, Ibrahim et al. [53] demonstrated that the log of Gross Domestic Product (GDP) per capita positively impacted the level of efficiency of the health system (β = 1.616). Also, the improvement of national economic indicators increases efficiency (*r* = 0.059) [15]. Although they did not directly measure national economic status and efficiency, Jordi et al. [16] believed that countries with robust economies with commensurate income are more likely to be efficient [16]. However, there is yet to be a conclusive discussion on the theoretical and empirical link pathway by which higher income leads to better levels of efficiency. For example, Eritrea is one of the most efficient countries in Africa, but Eritrea’s economy is weaker than many African economies [16]. Atake [43], a facility-level study, estimated that increasing the economic status of a country reduces inefficiency in both primary and curative healthcare settings (β = -0.074 to -0.022).

#### Type of facility

Obure et al. [47] reported that type of hospital was positively (*r* = 0.091) correlated with efficiency. A study conducted in Ethiopia [24] found that teaching hospitals were less efficient than other hospitals (*r* = 3.034). In contrast, Yitbarek et al. [23] found that higher levels of hospitals are positively correlated (*r* = 1.17) with efficiency, comparing secondary and primary hospitals.

#### Education index

The two studies [16,46] that examined the relationship between education and efficiency were conclusive that higher levels of education results in higher efficiency (*r* = 0.18 and *r* = 0.0249).

#### Hospital size

Three of the included studies [21,44,45] stated the relationship between hospital size and efficiency. Two of these studies [44,45] reported that bigger hospitals were significantly more efficient than smaller ones. Without stating the correlation coefficient, Marschall and Flessa [21] contrasted the findings of Ayiko et al. [44] and Mujasi et al. [45], demonstrating that hospitals that were too big were inefficient.

#### Healthcare expenditure share of GDP

Two studies [48,53] reported health and the relationship between healthcare expenditure as a share of GDP. Countries with higher healthcare expenditure as a share of GDP were likely more efficient than others. Ibrahim et al. [53] found a positive correlation between healthcare expenditure as a share of GDP and efficiency (β = 0.085). In the confirmation of the findings of Ibrahim et al. [53], See and Yen [48] reported that healthcare as a share of GDP and inefficiency had a negative correlation (β = -0.0399).

### Limitations of the included studies

Although poorly presented studies were eliminated during critical appraisal, below we present some limitations in the studies included in the analysis.

### Data unavailability or challenges

Data availability and quality concerns were the single most dominant limitation presented by the efficiency studies conducted in the WHO-Africa region. 14 studies [6,15,16,22,27,29-31,33,43,48-59] reported unavailability of data as limitations to their projections and methodological adjustments in their studies. For example, Top et al. [6] stated that lack of data on a comprehensive list of variables meant that limited variables were used for the modelling. Amponsah and Amanfo [31] and Alhassan et al. [55] could not estimate allocative efficiency due to data unavailability. Unavailability of data also influence the quality and completeness of input variables [6,15,27,29,45,48-50,54].

### Limited input variables

A limited number of input variables were used because data for other variables that could have influenced the efficiency scores were not available [15,23,25,27,35-37,40,43,49,50]. For example, Zarulli et al. [50] reported that a limited number of variables were used due to limited data availability. Anselmi [36] also reported that the lack of availability of data meant that the only input variable that was used was related to outpatients. Lastly, Bobo et al. [27] said that non-staff expenditures, such as costs related to pharmaceuticals and laboratory data, were not available to be used as inputs.

### Methodological limitations

Three studies [16,34,45] reported limitations in the data envelopment analysis, which might have influenced the efficiency scores of the countries and institutions they studied. As stated by Mujasi et al. [45], DEA is sensitive to measurement error, and the small sample size used in the study could mean that hospitals were more technically efficient than in reality. Omondi Aduda et al. [34] also reported that DEA assumes all errors are due to inefficiency, and its estimates are sensitive to outliers. Finally, Jordi et al. [16] stated that the deterministic nature of the data envelopment analysis method means that only available data can be used; as such, estimations were made beyond the available data.

## DISCUSSION

The concept of efficiency of health systems and its measurement is complex in that it depends on drivers, inputs and outputs that do not always share linear or well-defined relationships. The measurement processes are complicated because some of the inputs, such as household decisions, level of education, poverty and life expectancy, are outside the confines of the health system. Despite the intricacies of the concept and its measurement, estimating efficiency is crucial in ensuring the sustainability of health systems [31]. In recent global efficiency studies, African countries were found in the lowest bands of efficiency. Because many studies have reported varying efficiency figures for various countries within the WHO Africa region, it was necessary to synthesise these primary studies to produce a single efficiency score for the region.

### Strength of evidence used in the study

Synthesising 39 studies covering 46 out of the 47 (97.90%) countries within the WHO African region – except for South Sudan – this study provided a comprehensive quantitative and qualitative synthesis of the state of health system efficiency within the WHO-Africa region. Although there have been recent systematic reviews [5] on health system efficiency in Africa, this study provided the first pooled regional efficiency estimate using meta-analysis and country-specific estimates pooled from multicountry analyses. Efficiency studies are data intensive and might be difficult to carry out in South Sudan, where the health information systems may not be optimal [56]. Many efficiency studies were conducted in Ethiopia, Ghana, South Africa, Kenya and Uganda, many of which have been scored above 65% on the WHO score report, a global assessment of the status and capacity of health information systems [57]. Many studies cited data availability and quality as limitations to the depth of efficiency analysis. However, our robust and exhaustive search from all major databases and content aggregators ensured all the available studies within the African region were retrieved and synthesised to overcome the data limitation challenge. In particular, multicountry studies that included African countries provided data from the francophone and lusophone countries, mitigating the language limitation reported by many literature syntheses in the region.

### Methodological diversity in efficiency analyses: impact on our findings and suggestion towards harmonisation

The number of publications on efficiency in Africa have been increasing steadily since the last decade, demonstrating that efficiency is gaining much attention in both policy and intellectual discourse. However, this has come along with increasing methodological diversity in measuring health system efficiency. Although the majority of studies (69%, n = 27) employed DEA, which has been the mainstay research method in efficiency analysis, almost one-third (30.8%, n = 12) of studies used the SFA approach. Di Giorgio et al. [51] also argue that combining the two methodologies using the ensemble approach is gaining currency and could become the gold standard in future. Nevertheless, equally innovative approaches to efficiency analysis using non-parametric bootstrapping are being proposed and tested [43,48]. The ensuing diversity in the methodologies and the input variables used in the efficiency studies produce varying results for the same countries. Even within the same methodological approaches, such as DEA, we observed that there are vast differences in the selection of input and output variables for efficiency analysis, making comparability of results across studies complicated. Undoubtedly, these between-study differences impacted the level of heterogeneity of our pooled estimates of efficiency (*I^2^* = 98.48%). Thus, the pooled efficiency scores must be interpreted alongside contextual evidence on the factors that drive efficiency or the lack of it in a country. For example, two multicountry studies estimated the health system efficiency of Mauritius to be 14% in 2012 [14] and 78% in 2014 [13]. The sharp improvement in efficiency score by 64% within two years may not necessarily be a result of actual changes in the health system but differences in methods, inputs and outputs data used and the quality of that data.

Furthermore, we observed that part of the methodological diversity may also be linked to a lack of standardised reporting and quality appraisal tools for health system efficiency analysis. Thus, from a methodological perspective, it might be fruitful to begin a discourse towards developing these essential tools to harmonise reporting and quality assessment of health system efficiency analyses to ease interpretation and improve their utility in decision-making at the country level. In the absence of standardised tools for quality appraisal, we adapted a tool used in a previous systematic review [5] and added items borrowed from good practice tools in health economics modelling [58,59]. Our adapted quality checklist could be further refined through expert consensus or systematic research process towards a standard process of health system efficiency analysis.

### The level of efficiency in African health systems and the disparities

Using the random effects restricted maximum likelihood (REML) method, our pooled efficiency score for the Africa region was 0.77 (95% CI = 0.66-0.83). Thus, we estimate that the African region’s overall efficiency of health systems is about 77%, which could be between 66% and 83% when uncertainties in the data are considered. Our estimation of 23% (95% CI = 17-34) level of inefficiency is consistent with the World Bank’s estimate of 24% for Africa. The convergence of evidence between this study and that of the World Bank demonstrates the reliability of the analysis even when different approaches are used.

Additionally, the country-specific analysis in this study shows that the lowest level of efficiency was observed in Mauritius (55% efficient), while Eritrea was at the frontier (100% efficient), with eight countries (17.4%) being 90% efficient or better. About 72% of the countries had at least a 20% level of inefficiency. A study by the International Monetary Fund (IMF) using the SFA approach found that African countries had lower efficiency levels when compared with others globally. In the landmark study, Grigoli and Kapsoli [52] demonstrated that at 2009 spending levels, the levels of inefficiencies in African health systems could yield increased healthy life expectancy (HALE) by five years if they were adequately addressed by following best practices. Nonetheless, some African countries like Eritrea have consistently featured in most global analyses as an efficiency frontier. Thus, there are also positive lessons from the Africa region on health system efficiency that could be studied and leveraged by others.

Furthermore, our analysis found a systematic variation between the average scores from facility-level analysis and the system-level estimates. From 12 countries with studies reporting health facility-level analyses, their efficiency score was about 19% lower (ranging from 10% in Eritrea to 35% in Sierra Leone) compared to the multicountry system-wide studies. With limited data, this seeming systematic difference was difficult to investigate further within the scope of this study. However, drawing from the various limitations discussed across the studies, some plausible reasons may include data quality issues, time lag between data and the period of publication. In particular, multicountry studies mostly used the last year of publicly available data, which may sometimes be outdated. For example, Grigoli and Kapsoli [52] used 2009 expenditure data for analysis published in 2018. With these, it may well be that multicountry studies provided a significant overestimation of the level of efficiency in which the level of wastage reported will just be the tip of the iceberg. Alternatively, there could be moderating and mediating factors at the health system level that may not be the same at the health facility levels. For example, it is known that strong community health systems are sin qua non for high-achieving health systems in Africa, but facility-level studies may not capture the catalysing effect of community-based health interventions such as immunisation campaigns and public education, among others, on the health system efficiency. In that context, health facility-level analysis of efficiency would systematically overestimate the inefficiency of the health system (or underestimate the level of efficiency of the health system). Further work is therefore needed to establish the extent to which health facility efficiency drives the overall efficiency of the health system.

### Factors driving (in)efficiency in the African region

The major drivers of efficiency and inefficiency of health systems in the WHO Africa region include population density of hospital catchment area, governance, type of facility ownership, staff density, national economic status, type of facility, educational index, size of health facility, and duration of inpatient stay. Most of the major drivers significantly influence the efficiency of health systems and health facilities. There were some facility-level specific drivers such as waiting time, type of patient care and duration of operation of the facility. Likewise, some health systems specific drivers of efficiency include the Gini coefficient, scale efficiency and the happiness level of the population.

Although the studies were conducted in varying settings and levels within the health system, there seems to be a convergence of the key drivers of the health system and health facility efficiency. The extent (correlation coefficient) to which each of the variables drives efficiency or inefficiency varies from study to study, and the relationship (positive or negative) of associated drivers needs to be re-examined as there are some contradictions in the literature. These drivers could also be influenced by many factors outside the scope of the study. It is, therefore, necessary for researchers to pay critical attention to confounding variables when collecting and analysis efficiency data.

### Policy implications and lessons learnt

This section outlines some policy implications and key lessons learnt from the findings presented by the studies included in the analysis. With approximately 23% (17%-34%) of the inefficiency of health systems in Africa, improving efficiency alone will yield a maximum of 34% improvement in resource availability in Africa, assuming all countries are performing similarly to the frontier countries. However, with the low level of health expenditure per capita in Africa, the efficiency gains alone will likely be insufficient to meet the minimum requirement for universal health coverage. Improvement in the efficiency of health facilities is essential in freeing up funds for other health sector programmes, such as public health interventions [35], and to address equity gaps. There are two ways through which the efficiency of health systems could be improved. First, addressing existing inefficiencies and investing in areas with inadequate resources [41]. Second, efficiency could be improved by adopting long-term efficiency enhancement policies [49].

Governance plays a critical role in the efficiency of health systems [53]. The fact that countries with varied income levels performed efficiently means that any country can build an efficient health system [16]. This implies that how resources are managed is more important than the quantum of resources injected into health systems [6,53]. Training health facility leaders on the drivers of efficiency, such as waiting time at the health facilities, might improve the efficiency of the health systems [25]. The factors that were negatively associated with efficiency could be targeted and tackled by health policymakers to improve the efficiency in the various facilities [24]. The worst performing healthcare systems should learn from their peers on ways to improve the health system’s efficiency [16,42].

Decreasing the unemployment rate and income disparities in countries with low education levels would improve efficiency without increasing health expenditure [50], meaning that unemployment is a critical drive for inefficiency. The government should set up performance indicators and reward systems in the form of subsidies to encourage hospitals to be efficient [43]. The performance indicators need to be improved regularly to make judicious use of the scarce health resources [27].

Stakeholders may need to consider an effective resource allocation analysis [29,33], while health centres have been allocated more resources than required to produce their outcomes. There is a need to reallocate resources to use them to their maximum potential [28]. Routine data collection and monitoring are necessary for efficient health systems [22,26]. Also, incorporating service-quality dimensions and using stepwise multiple criteria in performance evaluation enhances the comprehensiveness and validity of the services rendered [34].

## CONCLUSIONS

Almost all the countries in the WHO-Africa region have reported efficiency studies, with the overall trend of publications in the area increasing, producing evidence on which policy and practice decisions could be based. We discovered a systematic variation between the average scores of facility-level and system-level efficiency estimates, with facility-level estimates about 19% lower (ranging from 10%-35%) than system-level studies.

With approximately 23% (17%-34%) of the inefficiency of health systems in Africa, improving efficiency alone will yield a maximum of 34%improvement in resource availability in Africa, assuming all countries are performing similarly to the frontier countries. However, with the low level of health expenditure per capita in Africa, the efficiency gains alone will likely be insufficient to meet the minimum requirement for universal health coverage. Lack of or incomplete data was cited as a critical limitation to the level and accuracy of the input and output variables examined in the studies.

Countries in Africa will need to build strong data capture and storage systems to improve the accuracy of research within the region. Therefore, a multi-country empirical study should be conducted to estimate the efficiency of health systems in Africa, paying attention to the major input/output variables, drivers and key lessons learnt in this review.

## Additional material


Online Supplementary Document
