# Supplementary material for: The state and significant drivers of health systems efficiency in Africa: A systematic review and meta-analysis
Source: J Glob Health. 2023 Nov 9;13:04131. doi: 10.7189/jogh.13.04131 (PMC10630696; doi:10.7189/jogh.13.04131)
Supplement: Online Supplementary Document [file jogh-13-04131-s001.pdf]

Table S1. Sample of the critical appraisal of studies included

Number	Criteria	Babalola and Moodley (2020)	Achoki et al. (2017)	Ayiko et al., (2020)	Jehu-Appiah et al (2014)	Kiddus et al., (2019)	Roberts et al., (2015)	See and Yen (2018)	Yitbarek et al., (2019)	Zeng et al. (2014)	Nassar et al., (2020)	Dayo et al., (2016)	Kinyanjui et al., (2015)	Anselmi et al., (2018)	Babalola, and Moodley (2020)	Kirigia and Asbu (2013)	Ali et al., (2017)	Jordi et al., (2020)	Top et al., (2019)
	**Objective and design**																		
1.	Was the objective(s) of the study clearly stated?	Y	Y	Y	Y	Y	Y	Y	Y	Y	Y	Y	Y	Y	Y	Y	Y	Y	Y
2.	Was the study design described?	Y	Y	Y	Y	Y	Y	Y	Y	Y	Y	Y	Y	Y	Y	Y	Y	Y	Y
3.	Was the study period defined?	Y	Y	Y	Y	Y	Y	Y	Y	Y	Y	Y	Y	Y	Y	Y	Y	Y	Y
4.	Was the scope of the study defined?	Y	Y	Y	Y	Y	Y	Y	Y	Y	Y	Y	Y	Y	Y	Y	Y	Y	Y
	**Sample**																		
5.	Was the sample size stated?	Y	Y	Y	Y	Y	Y	Y	Y	Y	Y	Y	Y	Y	Y	Y	Y	Y	Y
6.	Was the sample size justified?	Y	Y	Y	Y	NS	NS	NS	NS	Y	Y	NS	NS	Y	Y	Y	Y	Y	Y
7.	Was the sample representative of the defined scope of the study?	Y	Y	Y	Y	Y	Y	Y	Y	Y	Y	Y	Y	Y	Y	Y	Y	Y	Y
8.	Did the study include health facilities across the spectrum of healthcare delivery (example; public, private, teaching, health centre, primary/district hospital, etc)?	Y	Y	Y	Y	NS	NS	NS	NS	NS	NS	Y	NS	NS	NS	NS	NS	NS	HS
	**Indicators and data sources**																		
9.	Were the input indicators described?	Y	Y	Y	Y	Y	Y	Y	Y	Y	Y	Y	Y	Y	Y	Y	Y	Y	Y
10.	Were the input indicators justified?	Y	Y	Y	Y	Y	Y	Y	NS	NS	Y	NS	Y	Y	Y	Y	Y	Y	Y

11.	Were the data sources for input indicators transparently described?	Y	Y	Y	Y	NS	Y	NS	Y	Y	Y	Y	Y	Y	Y	Y	Y	Y	Y
12.	Were the output indicators described?	Y	Y	Y	Y	Y	Y	Y	Y	Y	Y	Y	Y	Y	Y	Y	Y	Y	Y
13.	Were the output indicators justified?	Y	Y	Y	Y	Y	Y	NS	NS	NS	Y	NS	Y	Y	Y	Y	Y	Y	Y
14.	Were the data sources for output indicators transparently described?	Y	Y	Y	Y	NS	Y	BS	Y	Y	Y	Y	Y	Y	Y	Y	Y	Y	Y
	**Statistical analysis**																		
15.	Were the statistical methods used adequately described?	Y	Y	Y	Y	Y	Y	Y	Y	Y	Y	Y	Y	Y	Y	Y	Y	Y	Y
16.	Was the statistical method justified?	Y	Y	Y	Y	Y	Y	Y	Y	Y	Y	Y	Y	Y	Y	Y	Y	Y	Y
	**Results, discussion, and conclusion**																		
17.	Did the results address the stated objectives of the study?	Y	Y	Y	Y	Y	Y	Y	Y	Y	Y	Y	Y	Y	Y	Y	Y	Y	Y
18.	Were the results interpreted accurately?	Y	Y	Y	Y	Y	Y	Y	Y	Y	Y	Y	Y	Y	Y	Y	Y	Y	Y
19.	Were the conclusions drawn from the results?	Y	Y	Y	Y	Y	Y	Y	Y	Y	Y	Y	Y	Y	Y	Y	Y	Y	Y
20.	Were the limitations of the study discussed?	Y	Y	Y	Y	Y	Y	Y	Y	Y	Y	Y	NS	Y	Y	Y	NS	Y	Y

Table S2: Data matrix

No.	Author, year<br><br>Country	Aim	Methodology	Sample	Types and scope	Input and output variables	Findings		Conclusions and limitations
							Efficiency level	Significant drivers and co-efficient and *p-value*	
**System wide studies**									
1.	Jordi et al., (2020)<br><br>Multiple countries	To examine the efficiency of the national health expenditure of countries across the world towards the attainment of Universal Health Coverage	Data envelopment analysis	172 countries worldwide consisting of 40 African countries	Technical efficiency and its drivers	Input: Current health expenditure<br><br>Output: Reproductive, maternal and child<br><br>health; non-communicable<br><br>diseases; communicable	The efficiency scores of African countries are as follows: Botswana (97%); South Africa (96%); Namibia (95%); Eswatini (92%); Kenya (94%); Malawi (94%); Morocco (87%); Zimbabwe (88%); Zambia (88%); Lesotho (86%); Libya (84%); Gabon (82%); Congo (84%); Mozambique (100%); and Equitorial Guinea (81%). Others include: Gambia (87%); Angola (77%); Ghana (77%); Ethiopia (85%); Tanzania	Governance capacity (r=0.027, p<0.05),<br><br>income and education (r=0.014, p<0.001) were significantly associated with<br><br>efficiency.	Conclusion: The worst performing healthcare systems should learn from their peer on ways to improve the health systems efficiency. Also, the fact that countries with varied income level performed efficiently means that any country can build an

No.	Author, year<br><br>Country	Aim	Methodology	Sample	Types and scope	Input and output variables	Findings		Conclusions and limitations
							Efficiency level	Significant drivers and co-efficient and *p-value*	
						diseases; service capacity and access; and current health expenditure.	(82%); Benin (80%); and Uganda (75%). The efficiency scores of the remaining African countries were: Rwanda (89%); Senegal (76%); Cameroon (71%); Cote d'Ivoire (70%); Liberia (71%); Guinea-Bissau (71%); Burkina Faso (73%); Guinea (78%); Democratic Republic of Congo (100%); Niger (74%); Sudan (62%); Chad (65%); Mauritania (59%); Central African Republic (100%); Sierra Leone (54%); and Mali (56%).		efficient health system.<br><br>Limitation: The deterministic nature of the data envelopment analysis method means that only available data can be used. As such, no estimations were made beyond the available data.

No.	Author, year<br><br>Country	Aim	Methodology	Sample	Types and scope	Input and output variables	Findings		Conclusions and limitations
							Efficiency level	Significant drivers and co-efficient and *p-value*	
2.	Kim and Kang (2014)<br><br>Multiple countries	To examine the technical efficiency of countries	Bootstrap data envelopment analysis	170 countries including 51 African countries	Technical efficiency within multiple countries	Inputs: Public health expenditure and schooling.<br><br>Outputs: Life expectancy at birth and the child mortality rate of<br><br>children under five.	Equatorial Guinea, Guinea, Niger, Morocco, Tunisia and Comoros were the only African countries that operated at 100% technical efficiency.		Conclusion: High income countries in African countries used their health resources better than other African countries.<br><br>Limitation: Contextual factors within each country may have affected the various inputs and outputs that were used.

No.	Author, year<br><br>Country	Aim	Methodology	Sample	Types and scope	Input and output variables	Findings		Conclusions and limitations
							Efficiency level	Significant drivers and co-efficient and *p-value*	
3.	Sinimole (2012)<br><br>Multiple countries	To examine the effectiveness of data envelopment analysis in measuring the efficiency of health systems across the world.	Data envelopment analysis	180 countries worldwide consisting of 13 African countries.	Technical efficiency among countries across the world, including African countries	Input: Density of all types of community health workers, external resources for health, general government expenditure on health, private expenditure on health, and proportion of population aged 15-24.	In terms of CRS scores, the technical efficiency of African countries included in the study was: Comoros (100%); Congo (84%); Eritrea (100%); Gambia (100%); Guinea (87%); Liberia (100%); Mauritius (14%); Morocco (10%); Sao Tome (68%); Seychelles (14%); Sierra Leone (87%); Tunisia (7%); and Liberia (100%). The VRS scores showed that all the African countries included in the study were 100% efficient.		Conclusion: Data envelopment analysis is effective in determining the efficiency of health systems.<br><br>Limitations: The data used for the study was from 2008, thus, newer data would present a more current picture on efficiency of health systems.

No.	Author, year<br><br>Country	Aim	Methodology	Sample	Types and scope	Input and output variables	Findings		Conclusions and limitations
							Efficiency level	Significant drivers and co-efficient and *p-value*	
						Output: Adult mortality rate, death due to various diseases, health life expectancy, number of reported cases of various diseases, antenatal care coverage, birth attended by skilled health personnel, children who received various			

No.	Author, year<br><br>Country	Aim	Methodology	Sample	Types and scope	Input and output variables	Findings		Conclusions and limitations
							Efficiency level	Significant drivers and co-efficient and *p-value*	
						immunisation doses, and tuberculosis treatment success rate.			
4.	Sun et al., (2017)<br><br>Multiple unspecified countries	To evaluate the efficiency of health systems among some 172 countries, including 43 African countries, worldwide	Data envelopment analysis technique using data retrieved from WHO and World Bank between 2004 to 2011	43 African countries	Technical efficiency in 43 African countries	Inputs: Health spending per capita<br><br>Output: Infant mortality rate, under 5 mortality rate, and life expectancy	The regional distribution of efficiency scores showed that Africa recorded the lowest efficiency of 67%. In 2011, many African countries including Equatorial Guinea, Botswana, South Africa, Swaziland, Gabon, Trinidad, Angola, and Lesotho were on the least of the countries with the poorest efficiency scores. The specific efficiency scores were not reported. On the other hands, Madagascar	National economic status (r=-0.059, p<0.001), HIV/AIDS prevalence (r=-0.059, p<0.001), health financing mechanisms (r=0.019, p<0.001), and	Conclusion: The findings demonstrate the importance of governance, health financing mechanisms, and the overall economic status of a country in enhancing efficiency of health systems.

No.	Author, year<br><br>Country	Aim	Methodology	Sample	Types and scope	Input and output variables	Findings		Conclusions and limitations
							Efficiency level	Significant drivers and co-efficient and *p-value*	
							ranked very highly from 2008 to 2011 with an efficiency score of 100%.	governance (r=0.044, p<0.01) were found to be statistically associated with the efficiency of national health systems.	Limitations: The only input variable used was health spending per capital. It was not possible to use other variables due to unavailability of data across all the countries. There was also missing data for 0.5% of the output variables. Rule of law was used to represent governance as a

No.	Author, year<br><br>Country	Aim	Methodology	Sample	Types and scope	Input and output variables	Findings		Conclusions and limitations
							Efficiency level	Significant drivers and co-efficient and *p-value*	
									driver of efficiency of health systems.
5.	Top et al., (2019)<br><br>Multiple countries	To measure and compare the health system efficiency of some 36 African countries	Data envelopment analysis	36 countries	Technical efficiency among African countries	Input: proportion of total health expenditures in the gross domestic product, the number of physicians and nurses, hospital beds per 1000 people, the unemployment rate, and the Gini coefficient.	Out of the 31 countries, the health systems of 21 (58.33%) were found to be efficient. These countries were Benin, Cabo Verde, Algeria, Chad, Ethiopia, Morocco, Gabon, Guinea, Liberia, Madagascar, and Malawi. The remaining countries that were found to have efficient health systems were Mali, Egypt, Mauritania, Nigeria, Niger, Rwanda, Senegal, Sierra Leone, Tanzania, and Tunisia. Regarding the countries with inefficient	Number of nurses per 1000 people (r=0.125, p<0.001), and Gini coefficient (r=0.018, p<0.001) variables statistically significantly affected the inefficiency of national healthcare systems.	Conclusion: Nations that wish to be at par with the healthcare systems of other nations must learn to make judicious use of health resources.<br><br>Limitations: The data that was used for the study was from 2010 to 2015, so caution must be taken when

No.	Author, year<br>Country	Aim	Methodology	Sample	Types and scope	Input and output variables	Findings		Conclusions and limitations
							Efficiency level	Significant drivers and co-efficient and *p-value*	
						Output: Life expectancy at birth and 1/(infant mortality rate).	health systems, the efficiency scores were: Botswana (64.3%); Burkina Faso (98.7%); Djibouti (90.3%); Gambia (95.4%); Ghana (93.5%); Guinea-Bissau (76.1%); South Africa (52.7%), Cameroon (87.5%); Kenya (90.4%); and Mozambique (85.5%). In addition, there were: Sudan (89.8%); Togo (90.6%); Uganda (90.6%); Zambia (74%); and Zimbabwe (84.7%).		attempting to generalize the result to current times. Also, lack of availability of data on a comprehensive list of variables meant that limited variables were used for the modeling.
**Facility/sub-national level studies**									
6.	Achoki et al. (2017)	To investigate the technical	Retrospective collection of data from	All 72 health districts	Technical efficiency, and	Input: Total Funds, medical personnel, and	The national average of technical efficiency was 61.5%. The worst performing	Maternal literacy was associated	The low national average of technical efficiency

No.	Author, year<br><br>Country	Aim	Methodology	Sample	Types and scope	Input and output variables	Findings		Conclusions and limitations
							Efficiency level	Significant drivers and co-efficient and *p-value*	
	Zambia	efficiency and its associated factors in the delivery of maternal and child health services	district-level databases		subnational and nation analysis.	nursing personnel.<br><br>Output: Under-5 mortality rate, under 5 survival rates.	district, Luangwa, had a technical efficiency of 31%, and the best performing district, Kafue, had a technical efficiency of 88%.	with technical efficiency (r=0.18, p<0.01)	means that more outcomes could be gotten from maternal and child health services without injecting extra resources.
7.	Aduda etl al., (2015)<br><br>Kenya	To assess the technical efficiency of voluntary medical male circumcision (VMMC)	Data envelopment analysis	21 health facilities	Technical efficiency among hospitals	Input: Clinicians, Nurse, Surgical Beds, Total Opening Time.<br><br>Output: MC performed, HTC performed,	The average technical efficiency scores under CRS, and VRS models<br><br>were 76%; and 84% in 2011 compared to 89%; and<br><br>89% in 2012	Unsatisfactory performance of tasks, lack of compliance to standard guidelines for service delivery. Inflexible obligatory	Conclusion: Incorporating service-quality dimensions and using stepwise-multiple criteria<br><br>in performance evaluation enhances

No.	Author, year<br>Country	Aim	Methodology	Sample	Types and scope	Input and output variables	Findings		Conclusions and limitations
							Efficiency level	Significant drivers and co-efficient and *p-value*	
						Quality of Service		institutional requirements and personnel factors and unexploited resources	comprehensiveness and validity of the services rendered.<br><br>Limitations: DEA assumes all errors are due to inefficiency and its estimates are sensitive to outliers.
8.	Alhassan et al., (2015)<br><br>Ghana	To examine the efficiency of facilities under the	Data envelopment analysis	64 facilities from 2 regions	Technical efficiency and its drivers	Input: Number of clinical staff, number of support staff, number of	The average technical efficiency of the facilities was 65%. 31% of the facilities attained 100% technical efficiency. Out of	Mission facilities (r= 52.1, p<0.001) and public	Conclusion: Facilities in urban areas wasted health resources. Stakeholders may

No.	Author, year<br><br>Country	Aim	Methodology	Sample	Types and scope	Input and output variables	Findings		Conclusions and limitations
							Efficiency level	Significant drivers and co-efficient and *p-value*	
		national health insurance scheme				beds, and number of detention wards.<br><br>Output: Number of deliveries, number of out-patients, number of antenatal and postnatal visits, and number of reproductive and family planning visits.	the facilities that recorded 100%, 10% were<br><br>mission/NGO; 40% were private-for-profit; 50 % were public/government.	facilities (r= 42.9,<br><br>p<0.05) had a positive, significant association with the technical efficiency. However, quality score per the NHIS accreditation data (r=−0.316, p<0.05) and	need to consider an effective analysis on resource allocation.<br><br>Limitation: The study did not include non-NHIS accredited facilities. Also, the study was conducted in only two out of the ten regions, so, the findings were not be generalizable to other regions. The

No.	Author, year<br><br>Country	Aim	Methodology	Sample	Types and scope	Input and output variables	Findings		Conclusions and limitations
							Efficiency level	Significant drivers and co-efficient and *p-value*	
								environmental safety for staff and patients (r=−0.2764, p<0.05) had a negative, significant association with technical efficiency.	study also failed to investigate allocative efficiency due to unavailability of data.
9.	Ali et al., (2017)<br><br>Ethiopia	To examine the technical efficiency and its associated factors	Data envelopment analysis	12 hospitals	Technical efficiency and its drivers among hospitals in Eastern Ethiopia between	Input: Beds, health staff and drug<br><br>Supplies.	The efficiency scores (VRS) showed that 6 (50%), 5 (42%), 3 (25%), 3 (25%), 4 (33%),<br><br>and 3 (25%) of the hospitals were technically inefficient between 2007/08 and 2012/13, respectively. The	Teaching hospitals are less efficient than other hospitals (r= 3.034, p<0.05). In	Conclusion: The factors that were negatively associated with efficiency could be targeted and tackled by health policy makers towards improving

No.	Author, year<br>Country	Aim	Methodology	Sample	Types and scope	Input and output variables	Findings		Conclusions and limitations
							Efficiency level	Significant drivers and co-efficient and *p-value*	
					2007/08 and 2012/13	Output: Outpatient visits, inpatient days, and surgery.	mean CRS TE score for 2007/08 was 78% and the mean VRS TE score was 83%. In 2008/09, the mean CRS TE score was 73% and the mean VRS TE score was 86%. In 2009/10, the mean CRS TE score was 83% and the mean VRS TE score was 93%.	addition, medical<br><br>doctor to total staff ratio (r=-26.65, $p<0.01$), the proportion of outpatient visit to inpatient days (r= −0.33, $p<0.1$), and the proportion of inpatients<br><br>treated per medical doctor (r= −4.81,	the efficiency in the various facilities.<br><br>Limitations:

No.	Author, year<br><br>Country	Aim	Methodology	Sample	Types and scope	Input and output variables	Findings		Conclusions and limitations
							Efficiency level	Significant drivers and co-efficient and *p-value*	
								p<0.1) were negatively related with technical inefficiency of hospitals	
10.	Amare et al., (2020)<br><br>Ethiopia	To assess the technical efficiency of maternal services in facilities based in the north-eastern part of the country	Data envelopment analysis	12 hospitals	Technical efficiency among public hospitals in 2011 fiscal years	Inputs: Salary expenditure, non-salary expenditure, and the number of beds.<br><br>Output: Antenatal care, skilled	58.3% hospitals were technically efficiency. The overall CRS score was 74% and the VRS score was 92%.	The hospital manager's experience year (r=0.031, p<0.05), the educational level of the manager (r=0.219, p<0.05), and the hospital service year (r=0.09,	Conclusion: Over half of the facilities were technically efficient. There is the need for enhanced monitoring and evaluation systems in the facilities so that any wasted resources would be

No.	Author, year Country	Aim	Methodology	Sample	Types and scope	Input and output variables	Findings		Conclusions and limitations
							Efficiency level	Significant drivers and co-efficient and *p-value*	
						delivery, and postnatal care		p<0.05) associated positively with the technical efficiency. However, the catchment population (r=-0.0524, p<0.05) and distance of another health facility (r=-0.479, p<0.05) associated negatively with technical efficiency.	injected into other healthcare services.<br><br>Limitation: The study only included public hospitals. Also, the study was only conducted using data from a single year, so the efficiency results might have changed over time.

No.	Author, year<br><br>Country	Aim	Methodology	Sample	Types and scope	Input and output variables	Findings		Conclusions and limitations
							Efficiency level	Significant drivers and co-efficient and *p-value*	
11.	Amponsah and Amanfo (2016)<br><br>Ghana	To examine the efficiency and growth of the maternal health systems in Ghana in relation to the Millennium Development Goals.	Data Envelopment Analysis-Malmquits Productivity<br><br>Index	10 regions	Technical efficiency and source of growth	Input: Number of doctors and nurses.<br><br>Output: Institutional maternal survival ratio.	Overall, the technical efficiency of the 10 regions increased by about 12.6%	The main source of growth in the maternal health systems was innovation.	Conclusion: Efficiency is crucial in ensuring the sustainability of health systems.<br><br>Limitations: Allocative efficiency was not determined due to unavailability of data on price information.
12.	Anselmi et al., (2018)<br><br>Mozambique	Examine the efficiency of delivery outpatient	Stochastic frontier analysis	133 districts	Technical efficiency in primary	Input: Health force staff, equipment index, total	The mean efficiency score for the facilities was 73%. None of the facilities was 100% efficient as the highest		Conclusion: The findings show that there is a need to improve the

No.	Author, year<br><br>Country	Aim	Methodology	Sample	Types and scope	Input and output variables	Findings		Conclusions and limitations
							Efficiency level	Significant drivers and co-efficient and *p-value*	
		services in primary healthcare facilities			health facilities	expenditure per capita, government district expenditure per capita, government provincial expenditure per capita, donor provincial expenditure per capita, government district expenditure per health force, and government	efficiency score was 96.8%, and the lowest efficiency score was 17.5%.		efficiency of health systems in the primary healthcare facilities. In order to do so, it may be necessary to study the drivers to efficiency and develop strategies to target those drivers.<br><br>Limitation: The lack of availability of data meant that the only input variable that was

No.	Author, year<br>Country	Aim	Methodology	Sample	Types and scope	Input and output variables	Findings		Conclusions and limitations
							Efficiency level	Significant drivers and co-efficient and *p-value*	
						provincial expenditure per health force.<br><br>Output: Outpatient consultations per capita per year			used related to outpatients.
13.	Atake (2019)<br>Togo	To analyse the technical efficiency of public hospitals	Double Bootstrap data envelopment analysis	139 public hospitals	Technical efficiency and its drivers	Inputs: Medical staff—all the medical doctors of all grades; paramedical staff—all the	For the small hospitals, 33.33%, 20.72%, and 14.41% were efficient in 2010, 2009, and 2008 respectively. Regarding the large hospitals, 17.85% were efficient 2010 and 2009 each,	For the small hospitals, per capita, per capita income (r=-0.074, p<0.001), hospital's balance (r=-	Conclusion: Government subsidies should be granted to hospitals when they meet certain performance indicators. This

No.	Author, year<br><br>Country	Aim	Methodology	Sample	Types and scope	Input and output variables	Findings		Conclusions and limitations
							Efficiency level	Significant drivers and co-efficient and *p-value*	
						nurses and midwives; technical staff—the laboratory technicians, their assistants, the resuscitators and anaesthetists; administrative staff—all those in charge of the administrative and financial management of the hospital; number of	and 28.57% were efficient in 2008.	0.004, $p<0.05$), density of medical staff ($r=-0.765$, $p<0.001$), density of paramedical staff ($r=-0.004$, $p<0.001$), density of technical staff ($r=-0.0926$, $p<0.001$), and competition ($r=-0.071$, $p<0.001$) were negatively	strategy would promote good hospital management and enhance the efficiency of the facilities.<br><br>Limitation: Less than one-third of the public hospitals in the country were included. So, the sample might not be representative. In addition inputs variables such

No.	Author, year<br><br>Country	Aim	Methodology	Sample	Types and scope	Input and output variables	Findings		Conclusions and limitations
							Efficiency level	Significant drivers and co-efficient and *p-value*	
						beds; and the number of diagnostic and special services provided by the facilities<br><br>Outputs: Number of people hospitalized weighted by the degree of severity of their illnesses; number of admissions to		associated with efficiency. For the small hospitals, hospital's balance (r=-0.0054, p<0.001), density of medical staff (r=-0.072, p<0.05), density of technical staff (r=-4.85, p<0.001), and competition (r=-0.041,	quality of equipment, staff absence, care quality, patient satisfaction was not included. These variables would allow for stronger results

No.	Author, year<br><br>Country	Aim	Methodology	Sample	Types and scope	Input and output variables	Findings		Conclusions and limitations
							Efficiency level	Significant drivers and co-efficient and *p-value*	
						hospitals; number of surgical operations; total number of birth deliveries; and total number of pregnant women seen during prenatal consultations.		p<0.001) were negatively associated with efficiency.	
14.	Ayiko et al., (2020)<br><br>Uganda	To examine the trend in technical efficiency of selected hospitals	Data envelopment analysis	78 hospitals (40 public hospitals and 38 private hospitals)	Technical efficiency among general hospitals		Overall, the average CRS TE score was 49% and the VRS TE score was 69%	Hospital size (r=0.31, p<0.05) geographical location (r=0.26, p<0.05),	It is crucial to consider the factors that determine efficiency of hospitals when making decisions on resource

No.	Author, year<br><br>Country	Aim	Methodology	Sample	Types and scope	Input and output variables	Findings		Conclusions and limitations
							Efficiency level	Significant drivers and co-efficient and *p-value*	
							The average technical efficiency in all the hospitals remained unstable from 2012 to 2017. In 2012/13, the average technical efficiency was 50%. This increased to 53% in 2014/2015, afterwards, it decreased to 49% in 2016/17. The technical efficiency of private hospital remained consistently higher than the technical efficiency of public hospitals. In 2012/13, the average technical efficiency of private hospitals was 71.5% whilst that of public hospitals was 63%. In 2014/15, the average	training status (r=0.31, p<0.05), and average days of in- patient stay (r=0.19, p<0.01) were statistically significant determinants of hospital efficiency	allocation to hospitals.

No.	Author, year<br><br>Country	Aim	Methodology	Sample	Types and scope	Input and output variables	Findings		Conclusions and limitations
							Efficiency level	Significant drivers and co-efficient and *p-value*	
							technical efficiency of private hospitals was 70% whilst that of public hospitals was 69%, and in 2016/17, the average technical efficiency of private hospitals was 73% whilst that of public hospitals was 64%.		
15.	Babalola, and Moodley (2020)<br><br>South Africa	To assess the technical efficiency of district hospital in the KwaZulu-Natal province	Data envelopment analysis	All the 38 public district hospitals in the province	Technical efficiency among public district hospitals from 2014 to 2017 in the KwaZulu-Natal province	Input: Medical and dental personnel (doctors and dentists),<br><br>nursing personnel (nurses and midwives),	Out of the 38 hospitals, those that were technically efficient<br><br>according to constant return to scale were 12 (31.6%), 16<br><br>(42.1%) and 14 (36.8%) in 2014/15, 2015/16 and 2016/17 respectively		Conclusion: A substantial proportion of the hospitals were technically efficient. This requires measures that are aimed at improving the utilization of the

No.	Author, year<br><br>Country	Aim	Methodology	Sample	Types and scope	Input and output variables	Findings		Conclusions and limitations
							Efficiency level	Significant drivers and co-efficient and *p-value*	
						pharmacy personnel<br><br>(pharmacists and pharmacist assistants), allied personnel (laboratory<br><br>scientist/technicians, radiographers, physiotherapists, etc.),<br><br>support/other services personnel	while according to the variable return to scale, the technically<br><br>efficient facilities were 22 (57.9%), 19 (50.0) and 21<br><br>(55.2%) respectively in 2014/15, 2015/16 and 2016/17 respectively. Overall, 23.7% of the hospitals were operating at optimal<br><br>scale of service delivery during the course of this study.		services of district public hospitals.<br><br>Limitations: Other categories of hospitals such as private and non-profit hospitals were not included in the study, so, the results may not be generalizable to the entire health systems at the district level. Also, data on the severity of the illnesses

No.	Author, year Country	Aim	Methodology	Sample	Types and scope	Input and output variables	Findings		Conclusions and limitations
							Efficiency level	Significant drivers and co-efficient and *p-value*	
						(social workers, cleaners, maintenance etc.) and number of beds.<br><br>Output: Total inpatient days, total outpatient headcount, total<br><br>theatre/operation cases, X-rays done,			treated were not available.

No.	Author, year<br>Country	Aim	Methodology	Sample	Types and scope	Input and output variables	Findings		Conclusions and limitations
							Efficiency level	Significant drivers and co-efficient and *p-value*	
						delivery by caesarean, and<br>regular delivery.			
16.	Bobo et al., (2018)<br>Ethiopia	To measure the technical efficiency of public health centres	Data envelopment analysis	16 health centres from 3 districts	Technical efficiency and its drivers	Input: Clinical staff, and non-clinical staff<br>Output: Outpatients visits, pentavalent 3 times, ANC visits for more than four times, delivery, and	The CRS score for technical efficiency was 77% and the VRS score for technical efficiency was 94%. 8, representing 50% of the health centres included, were 100% technically efficient.	Catchment population (r= 7.80E−06, p<0.05) and number of clinical staff (r=0.06, p<0.001) were found to be directly associated with efficiency. However the	Conclusion: Performance indicators have to be improved so as to make judicious use of the scare health resources.<br>Limitations: The data for the study was from the 2013/14 year, so,

No.	Author, year<br><br>Country	Aim	Methodology	Sample	Types and scope	Input and output variables	Findings		Conclusions and limitations
							Efficiency level	Significant drivers and co-efficient and *p-value*	
						family planning.		number of nonclinical staff was found to be inversely associated with efficiency (r=-0.03, p<0.05).	the findings of the study may not be applicable to the current age. Also, non-staff expenditure such as costs related to pharmaceuticals and laboratory data were not available to be used as inputs.
17.	Di Giorgio et al., (2016)<br><br>Multi-countries	To determine the technical efficiency of facilities in Kenya, Uganda, and Zambia	Combined restricted versions of data envelopment analysis and stochastic	395 facilities	Technical efficiency	Input: doctors, nurses, other staff, Non-medical staff and beds	The average efficiency scores in each of the three countries was below 50%. Kenya (34%), Uganda (40%), and Zambia (39%).		Conclusion: The number people receiving antiretroviral therapy could be improved if efficiency of the

No.	Author, year<br><br>Country	Aim	Methodology	Sample	Types and scope	Input and output variables	Findings		Conclusions and limitations
							Efficiency level	Significant drivers and co-efficient and *p-value*	
			distance function			Output: Outpatient visits , ANC visits, ART visits, Inpatient visits and deliveries			facilities are enhanced.<br><br>Limitation: The data across the three countries varied. So, even though measures were taken to make sure that the data were comparative, certain contextual factors such as the quality of medical staff, and medical services could have led to inherent

No.	Author, year<br><br>Country	Aim	Methodology	Sample	Types and scope	Input and output variables	Findings		Conclusions and limitations
							Efficiency level	Significant drivers and co-efficient and *p-value*	
									differences between the data.
18.	Grigoli and Kapsoli (2017)<br><br>Multi-countries	To estimate the technical efficiency of developing countries	Stochastic frontier analysis	64 countries worldwide consisting of 27 African countries.	Technical efficiency among countries across the world, including African countries	Input: Health expenditure per capita,<br><br>Output: Health adjusted life years, life expectancy, mortality under 5 years, infant mortality, maternal mortality, TB treatment success rate,	The technical efficiency scores were: Togo (97.4%); Namibia (97.4%); Benin (94.8%); The Gambia (94.6%); Tunisia (94.6%); Mauritius (94.5%); Botswana (94.4%); Malawi (94.4%); Mozambique (96.7%); and Liberia (96.7%). The others included Morocco (96.1%), Kenya (96.1%), Gabon (95.3%), Tanzania (95.3%), Cote d'ivoire (95.1%), and Congo DR (94.8%). In addition, there was Senegal (93.8%), Central African Republic		Conclusion: African country had the lowest efficiency scores. African countries have the potential to boost their average life expectancy by 5 years if they adopt standard operation measures in running their health systems.

No.	Author, year<br><br>Country	Aim	Methodology	Sample	Types and scope	Input and output variables	Findings		Conclusions and limitations
							Efficiency level	Significant drivers and co-efficient and *p-value*	
						measles immunization rate, polio immunization rate, and diphtheria, pertussis and tetanus immunization rate.	(93.7%), Ghana (93.6%), South Africa (92.9%), Cameroon (91.8%), Uganda (91.3%), Rwanda (91.1%), Swaziland (90.3%), Mali (89.8%), Zambia (89.2%), Lesotho (89.2%), and Sierra Leone (81%).		Limitations:
19.	Ibrahim et al., (2018)<br><br>Multiple countries	To assess the technical efficiency of health systems in SSA and rank the countries according to	Data envelopment analysis	39 countries	Technical efficiency and its drivers among countries in SSA	Input: Health expenditure per capita, immunized measles, immunized diphtheria-pertussis-tetanus, and	The overall health systems in SSA are inefficient. The median technical efficiency scores across all the SSA countries within the study period are as follows: 70.0% in 2010; 72.5% in 2011; 75% in 2012; 76% in 2013; 77% in 2014; and 78% in 2015. At	The factors that were positively associated with technical efficiency included: scale efficiency (r=0.3,	Conclusion: The findings showed that governance measures had stronger impact on efficiency of health systems than expenditure on public health. This

No.	Author, year<br><br>Country	Aim	Methodology	Sample	Types and scope	Input and output variables	Findings		Conclusions and limitations
							Efficiency level	Significant drivers and co-efficient and *p-value*	
		their technical efficiency.				immunized hepatitis B.<br><br>Output: Life expectancy, infant mortality rate, maternal mortality rate, tuberculosis rate, newly infected HIV, and malaria cases reported.	the national level, the health systems of only three countries were efficient in the duration of the study. These were Botswana in 2015, Tanzania in 2015, and Rwanda in 2014 and 2015. In 2010, 17 countries scored less than 50% at technical efficiency. These were: Angola; Burkina Faso; Burundi; Chad; Democratic Republic of Congo; Cote d'ivoire; Eritrea; Guinea; Guinea Bissau; Mali; Mozambique; Niger; Nigeria; Swaziland; Togo; Uganda; and Zimbabwe. In 2011, 18 countries had	p<0.01); log of GDP per capita (r=1.616, p<0.01); urbanisation (r=0.119, p<0.01); rule of law (r=0.294, p<0.001); government effectiveness (r=0.2, p<0.001); and public health expenditure (r=0.085, p<0.01).	implies that the manner in which resources are managed has more important than the quantum of resources that is injected into health systems.<br><br>Limitations:

No.	Author, year<br><br>Country	Aim	Methodology	Sample	Types and scope	Input and output variables	Findings		Conclusions and limitations
							Efficiency level	Significant drivers and co-efficient and *p-value*	
							technical efficiency scores below 50%. These were: Angola; Burkina Faso; Benin; Burundi; Chad; Democratic Republic of Congo; Cote d'ivoire; Cameroon; Guinea; Guinea Bissau; Mali; Mozambique; Niger; Liberia; Swaziland; Togo; Uganda; and Zimbabwe. In 2012, 17 countries were less than 50% technically efficient. These countries included: Angola; Burkina Faso; Burundi; Chad; Democratic Republic of Congo; Central African Republic; Cote d'ivoire; Benin; Guinea; Guinea		

No.	Author, year<br><br>Country	Aim	Methodology	Sample	Types and scope	Input and output variables	Findings		Conclusions and limitations
							Efficiency level	Significant drivers and co-efficient and *p-value*	
							Bissau; Mali; Mozambique; Niger; Liberia; Swaziland; Togo; Uganda; and Zimbabwe. In addition, there were 19 countries in 2013 who had less than 50% technical efficiency scores. These countries were: Angola; Burkina Faso; Burundi; Chad; Democratic Republic of Congo; Cameroon; Central African Republic; The Gambia; Cote d'ivoire; Benin; Guinea; Guinea Bissau; Mali; Mozambique; Niger; Liberia; Swaziland; Togo; Uganda; and Zimbabwe. In furtherance, 20 countries		

No.	Author, year<br><br>Country	Aim	Methodology	Sample	Types and scope	Input and output variables	Findings		Conclusions and limitations
							Efficiency level	Significant drivers and co-efficient and *p-value*	
							were less than 50% technically efficient in 2014. These were: Angola; Burkina Faso; Burundi; Chad; Central African Republic; Democratic Republic of Congo; Cote d'ivoire; Benin; Equatorial Guinea; The Gambia; Guinea; Guinea Bissau; Mali; Mozambique; Niger; Nigeria; Swaziland; Togo; Uganda; and Zimbabwe. Finally, in 2015, 20 countries had less than 50% technical efficiency in 2015. These countries were: Angola; Burkina Faso; Burundi; Chad; Democratic Republic of Congo; Cote		

No.	Author, year<br>Country	Aim	Methodology	Sample	Types and scope	Input and output variables	Findings		Conclusions and limitations
							Efficiency level	Significant drivers and co-efficient and *p-value*	
							d'ivoire; Benin; Cameroon; Central African Republic; Equatorial Guinea; Senegal; Guinea; Guinea Bissau; Mali; Mozambique; Niger; Nigeria; Swaziland; Togo; Uganda; and Zimbabwe.		
20.	Ichoku et al., (2014)<br>Nigeria	To determine the technical efficiency and its determinants in hospitals in south-eastern Nigeria	Stochastic frontier analysis	187 hospitals	Technical efficiency and its determinants	Inputs: The number of admissions, the number of<br>outpatients, the number of X-rays conducted at the<br>X-ray department if	The mean efficiency score was 71%. The minimum efficiency score was 26% and the maximum efficiency score was 94%. The mean efficiency score of private hospitals was 75% whilst the mean efficiency score of public hospitals was 58%.	Ownership was a driver of efficiency as privately owned hospitals were significantly more efficient than publicly owned hospitals.	Conclusion: The performance of the health systems in the study area could be attained by investing only 70% of the resources. This means that there is a substantial waste of health investments. The public

No.	Author, year<br><br>Country	Aim	Methodology	Sample	Types and scope	Input and output variables	Findings		Conclusions and limitations
							Efficiency level	Significant drivers and co-efficient and *p-value*	
						this existed and the medical<br><br>laboratory of the hospitals, recurrent costs, and capital costs.<br><br>Outputs:			hospitals in particular waste more resources. Studies should be carried out to understand the causes of the wastes in order to address these wastages.
21.	Ichoku et al., (2011)<br><br>Nigeria	To estimate the technical efficiency of health facilities in South-	Data envelopment analysis	200 hospitals	Technical efficiency	Inputs: Number of different categories of staff, the number of beds and recurrent	CRS technical efficiency score was 59% and VRS efficiency score was 72%.		Conclusion: There is a huge potential to improve the efficiency of health facilities within the south-eastern part of Nigeria, and by

No.	Author, year<br><br>Country	Aim	Methodology	Sample	Types and scope	Input and output variables	Findings		Conclusions and limitations
							Efficiency level	Significant drivers and co-efficient and *p-value*	
		eastern Nigeria				costs of services as well as capital costs such as building, expenditures on electric generators, and vehicles.<br><br>Outputs: Number of admissions, the number of outpatients, the			so doing, increasing the health outcome of the population. Improving efficiency would improve the socio-economic condition of people.

No.	Author, year<br><br>Country	Aim	Methodology	Sample	Types and scope	Input and output variables	Findings		Conclusions and limitations
							Efficiency level	Significant drivers and co-efficient and *p-value*	
						number of X-rays done, and the medical laboratory of the hospitals.			
22.	Jarue et al., (2015)<br><br>Gambia	To examine the technical efficiency across secondary level health facilities in the country	Data envelopment analysis	41 health centres	Technical efficiency among secondary health facilities	Input: Number of clinical staff, number of non-clinical staff, and number of beds.<br><br>Output: Total number of inpatients admitted, and total number of	The mean VRS technical efficiency score was 64.8%. The minimum efficiency score was 28%. Only 22% of the facilities included in the study were 100% technically efficient.		Conclusion: Health centres have been allocated more resources than they require to produce their outcomes. There is the need to re allocate resources in order to make maximum use of the resources.

No.	Author, year<br><br>Country	Aim	Methodology	Sample	Types and scope	Input and output variables	Findings		Conclusions and limitations
							Efficiency level	Significant drivers and co-efficient and *p-value*	
						outpatients treated and discharged.			
23.	Jehu-Appiah et al (2014)<br><br>Ghana	To compare the technical efficiency of health facilities according to ownership	Data envelopment analysis	Total number of 128 hospitals including 73 public hospitals, 42 mission hospitals, 7 quasi-government hospitals and 6 private hospitals	Technical efficiency and its drivers among health facilities	Input: total recurrent expenditures, number of clinical staff, number of nonclinical staff, and number of beds.<br><br>Output: annual total of outpatient	70.35% of public hospitals were technically efficient, 68.59% of mission hospitals were technically efficient, 55.83% of private hospitals were technically efficient, and 83% of quasi-government hospitals were technically efficient. In ascending order of increasing, the technical efficiency scores according to regions were Volta (45.02%); Upper West (50.65%); Easter (54.61%); Ashanti (61.99%); Brong	Quasi-government ownership is positively associated with hospital technical efficiency (r=22.514, p<0.05), private ownership negatively affects hospital efficiency (r=-	Conclusion: Ownership type drives disparities in terms of technical efficiency in health facilities.<br><br>Limitations: The data used for the study was based on figures from 2005 so the findings may not be accurately representing the

No.	Author, year<br><br>Country	Aim	Methodology	Sample	Types and scope	Input and output variables	Findings		Conclusions and limitations
							Efficiency level	Significant drivers and co-efficient and *p-value*	
						visits, inpatient days, deliveries, laboratory test.	Ahafo (62.04%); Greater Accra (65.04%); Western (70.01%); Upper East (72.23%); Central (76.98%); and Northern (83.36%).	23.782, p<0.01).	current situation on efficiency in the health facilities. Also, pharmaceuticals and non-pharmaceutical supplies were not included as input variables because such data was not available. The study also failed take into consideration differences between the categories of nurses and doctors in the

No.	Author, year<br><br>Country	Aim	Methodology	Sample	Types and scope	Input and output variables	Findings		Conclusions and limitations
							Efficiency level	Significant drivers and co-efficient and *p-value*	
									various hospitals. Even in the event that some hospitals had similar categories of nurses and doctors, the individual level of commitment, skills, and competencies may vary.
24.	Kinfu (2013)<br><br>South Africa	To determine the efficiency of health systems	Stochastic frontier analysis	52 districts	Technical efficiency of health systems in districts across the country	Input: Per-capita public expenditures on health, health<br><br>insurance coverage, the	The mean technical efficiency score of 81%. 8 of the 52 districts in the country had an efficiency score of below<br><br>60%, and 4 of these, the score was below 50%. The technical efficiency scores,		Conclusion: There are two ways through which efficiency of health systems could be improved: addressing existing inefficiencies and investing in areas

No.	Author, year<br><br>Country	Aim	Methodology	Sample	Types and scope	Input and output variables	Findings		Conclusions and limitations
							Efficiency level	Significant drivers and co-efficient and *p-value*	
						proportion of the population<br><br>with access to safe drinking water, sanitation and<br><br>waste disposal, the density of hospital beds and the<br><br>number of health workers in a district.	according to provinces, are as follow: Eastern Cape (82.5%); Free State (75%); Gauteng (91.5%); KwaZulu-Natal (59.9%); and Limpopo (96.7%). The others include Mpumalanga (63.3%), Northern Cape (84.8%), North West (80.1%), and Western Cape (99.0%).		with inadequate resources.<br><br>Limitation:

No.	Author, year<br>Country	Aim	Methodology	Sample	Types and scope	Input and output variables	Findings		Conclusions and limitations
							Efficiency level	Significant drivers and co-efficient and *p-value*	
						Outputs: Under-five mortality and coverage of birth<br><br>care (or institutional delivery rates).			
25.	Kinyanjui et al., (2015)<br><br>Kenya	To determine the efficiency of hospitals belong to faith-based organisations	Data envelopment analysis	30 hospitals in total which comprised of 10, 19 and 1 hospitals from the Christian Health	Technical efficiency of faith-based organisations in Kenya	Input: Number of medical officers and medical specialists, number of nurses in individual hospitals, number of beds	The mean CRS TE score was 58.8%, and the mean VRS TE score was 77.9%.<br><br>Out of all the hospitals, 11 (36.67%) were technically efficient i.e. (they scored 100% for technical		Conclusion: There are high levels of inefficiencies in the faith-based organization hospitals. If the hospitals were to operate as a group, 79% of all faith-based hospitals

No.	Author, year<br><br>Country	Aim	Methodology	Sample	Types and scope	Input and output variables	Findings		Conclusions and limitations
							Efficiency level	Significant drivers and co-efficient and *p-value*	
				Association, Conference of Catholic Bishops and the Supreme Council Muslims, respectively.		and cots in an individual facility, and other hospital workers.<br><br>Output: Inpatient and outpatient numbers	efficiency. Out of the technical efficient hospitals, 9 (81.82%) belonged to the Kenya Conference of Catholic Bishops while only 2 (18.18%) belonged to the Christian Health Association of Kenya. Meanwhile, the lowest score for technical efficiency was 28.4%.		could operate at 100% efficiency.<br><br>Limitations:
26.	Kirigia and Asbu (2013)<br><br>Eritrea	To assess technical efficiency and its determinants in public	Data envelopment analysis	19 hospitals	Technical efficiency and its drivers in public secondary	Input: Number of doctors, number of nurses and midwives; number of laboratory	The mean CRS TE score was 90.3% and the VRS TE score was 96.9%.	Average length of stay had a positive association with efficiency (r=0.1552) whilst	Conclusion: The findings showed that routinely collected data is useful in understanding efficiency of health

No.	Author, year<br><br>Country	Aim	Methodology	Sample	Types and scope	Input and output variables	Findings		Conclusions and limitations
							Efficiency level	Significant drivers and co-efficient and *p-value*	
		secondary hospitals			community hospitals	technicians; and number of operational beds and cots.<br><br>Output: Number of inpatient department visits and number of inpatient department discharges	8 (42%) of the hospitals were technically efficient while 11 (58%) were not technically efficient. Out of the hospitals that were not technically efficient, 6 of the hospitals were 91-99% efficient, 2 were 81-90% efficient, another 2 were 71-80% efficient, and 1 was<br><br>less than 71% efficient. The lowest efficiency score was 27.9%, and the mean efficiency score for all the included hospitals was 90.3%.	outpatients visit as a proportion of inpatient stays had a negative association with efficiency ($r=-1.333$). Each of the two variables was statistically significant ($p<0.05$).	systems towards enhancement of the performance of health facilities.<br><br>Limitation: The data was collected in 2007, and thus, the findings may not represent the current situation. Also, data was unavailable on pharmaceutical expenditure.

No.	Author, year<br>Country	Aim	Methodology	Sample	Types and scope	Input and output variables	Findings		Conclusions and limitations
							Efficiency level	Significant drivers and co-efficient and *p-value*	
27.	Kirigia et al., (2011)<br>Sierra Leone	To assess the technical efficiency of samples of community health centres, community health posts and maternal and child health posts	Data envelopment analysis	36 maternal and child health posts, 22 community health centres and 21 community health posts	Technical efficiency in two districts	Inputs: clinical staff and support staff.<br>Outputs: Outpatient, maternal, child health and family planning visits and plus immunization visits.	Regarding the maternal and child health posts, average score for CRS technical efficiency was 42.7%, and VRS technical efficiency the average score was 68.2%. Pertaining to the community health centres, CR was 62.4%, and VRS was 69.2%. The CRS technical efficiency score for community health posts was 57.2% and VRS score was 59%.		Conclusion: Substantial amount of wastage in the health systems were present. There is the need for routine monitoring of investments into health facilities and the performance indicators to inform decisions on resource allocation.<br>Limitations: Some of variables on health status that

No.	Author, year<br><br>Country	Aim	Methodology	Sample	Types and scope	Input and output variables	Findings		Conclusions and limitations
							Efficiency level	Significant drivers and co-efficient and *p-value*	
									were used in the study could have been influenced by the use of the health facilities.
28.	Marschall and Flessa (2011)<br><br>Burkina Faso	To determine the technical efficiency of a health facility within a rural setting	Data envelopment analysis	24 health facilities	Technical efficiency and its drivers	Inputs: Personnel cost, area [m2] , equipment, depreciation, and vaccine<br><br>Outputs: Number of general consultations, number of	The CRS scores for the facilities included from the four sub-districts included were: Sono (57.6%); Toni (79.1%); Werebere (84.9%); and Yevedougou (24.5%). The VRS scores for the facilities included from the four sub-districts included were: Sono (97.6%); Toni (94%); Werebere (99.9%); and Yevedougou (88.2%).	Efficiency was due to the poor utilization of the facilities—the facilities were either too big or they were serving areas with low population (low demand)	Conclusion: Policy makers must design healthcare services with the demand in mind.<br><br>Limitation: The source of the data that was used for the study did not collect data on certain variables

No.	Author, year<br><br>Country	Aim	Methodology	Sample	Types and scope	Input and output variables	Findings		Conclusions and limitations
							Efficiency level	Significant drivers and co-efficient and *p-value*	
						deliveries, number of other care, and number of vaccinations.			that were included in the study. Also, the study did not factor in the quality of health services they were rendered
29.	Mujasi et al., (2016)<br><br>Uganda	To evaluate the technical efficiency of referral hospitals	Data envelopment analysis	18 hospitals including 14 public sector regional referral and 4 large private not for profit hospitals	Technical efficiency and its drivers among referral hospitals for the 2012/13 fiscal year	Input: Total number of health workers and total number of hospital beds.<br><br>Output: Outpatient visits,	The mean CRS score was 79.4% and the mean VRS score was 91.4%. Only 18% of the hospitals were CRS technically efficient, and 47% were purely technically efficient.	The drivers of technical efficiency were: hospital size (r= -0.317, p<0.01); bed occupancy rate (r=-0.01, p<0.01) and outpatient visits as a	Conclusion: Most of the hospitals were inefficient, and this ought to be a concern for policy makers. The hospitals with the lowest efficiency scores should be investigated for further insight into the situation.

No.	Author, year<br><br>Country	Aim	Methodology	Sample	Types and scope	Input and output variables	Findings		Conclusions and limitations
							Efficiency level	Significant drivers and co-efficient and *p-value*	
						Deliveries and Inpatient days		proportion of inpatient<br><br>days (r=-0.095), p<0.01).	Limitation: DEA analysis is sensitive to measurement error, and the small sample size used in the study could mean that hospitals were more technically efficient than in reality. Also, there was no data on pharmaceuticals and laboratories. So they were not used as part of the variables. The lack

No.	Author, year<br><br>Country	Aim	Methodology	Sample	Types and scope	Input and output variables	Findings		Conclusions and limitations
							Efficiency level	Significant drivers and co-efficient and *p-value*	
									of data on prices also meant that it was not possible to estimate allocative efficiency.
30.	Nassar et al., (2020)<br><br>Multiple countries with 2 African countries (South Africa and Algeria)	To determine the technical efficiency of the healthcare systems of 21 middle income countries worldwide, including countries from Africa. The study	Stochastic Frontier Analysis	2 countries (South Africa and Algeria)	Technical efficiency in South Africa and Algeria from 2000 to 2017	Input: Current health expenditure as<br><br>a percentage of GDP, out-of-pocket expenditure per capita at purchasing power parity,<br><br>food production	South African recorded the lowest total technical efficiency score among all the 21 countries sampled from across the world. The efficiency of South African during the period of the study was 71%. South Africa also had the biggest gap between transient efficiency and persistent efficient. The transient efficiency score was 100% whilst the persistent efficiency score was 71%.		Conclusion: The low persistent efficiency score of South Africa means that efficiency could be improved by adopting long term efficiency enhancement policies.

No.	Author, year Country	Aim	Methodology	Sample	Types and scope	Input and output variables	Findings		Conclusions and limitations
							Efficiency level	Significant drivers and co-efficient and *p-value*	
		also sought to compare the transient efficiency scores and the persistent efficiency scores of the various countries.				index, current GDP per capita at purchasing power parity, inflation rate, education index, and urbanization.<br><br>Output: Life expectancy	Meanwhile, Algeria recorded 100% transient efficiency score, and 97% persistent efficiency score. Algeria also had an overall efficiency score of 98% during the duration of the study.		Limitation: There was missing data for some of the input variables which could have led bias in the results. Also, the input variables were proxies to represent social, economic, and environmental factors. A complete set of variables were not used in the analysis.
31.	Ngobeni et al., (2020)	To determine the technical efficiency of	Data envelopment analysis	*All the 9 provinces	Technical efficiency	Input: Health staff, and total	The mean technical efficiency scores ranging		Conclusion: Thus, inefficient provinces could

No.	Author, year<br><br>Country	Aim	Methodology	Sample	Types and scope	Input and output variables	Findings		Conclusions and limitations
							Efficiency level	Significant drivers and co-efficient and *p-value*	
	South Africa	the public health systems across all the provinces				health spending<br><br>Output: Infant mortality ratio	from 35.7% to 87.2% between the health.<br><br>Gauteng province had the technical efficiency. The second-best performing<br><br>province was the North West province. Other provinces like KwaZulu-Natal, Limpopo and the Eastern Cape only perform<br><br>well under the VRS. Meanwhile, the other three provinces are inefficient.		improve the use of inputs within a range of 64.3% and 20.8%.<br><br>Limitations: The study failed to analyse the drivers of technical efficiency

No.	Author, year<br>Country	Aim	Methodology	Sample	Types and scope	Input and output variables	Findings		Conclusions and limitations
							Efficiency level	Significant drivers and co-efficient and *p-value*	
							Overall, when total health expenditure and infant mortality were used as variables, the mean CRS TE score was 35.7% and the mean VRS TE score was 63.9%. When health staff and infant mortality were used as variables, the mean CRS TE score was 35.4% and the mean VRS TE score was 69.0%. And when , total health expenditure, health staff and infant mortality were used as variables, the mean CRS TE score was 63.9% and the mean VRS TE score was 87.2%.		

No.	Author, year<br><br>Country	Aim	Methodology	Sample	Types and scope	Input and output variables	Findings		Conclusions and limitations
							Efficiency level	Significant drivers and co-efficient and *p-value*	
32.	Novignon and Nonvignon (2017)<br><br>Ghana	To measure the efficiency of primary healthcare facilities and investigate the difference in efficiency of private and public facilities	Stochastic Frontier Analysis	87 health centres	Efficiency among primary health facilities	Input: number of personnel, hospital beds, expenditure on other capital items and administration<br><br>Output: Outpatient visits	The median efficiency score for the primary health centres was 51%. The average efficiency scores for private and public facilities were 65% and 50% respectively.		Conclusion: The general levels of efficiency in primary health facilities were poor. There were also disparities in the efficiency of the health facilities.<br><br>Limitations: No sensitivity analysis was conducted. Also, there was lack of data on certain expenditure. In addition, the

No.	Author, year<br><br>Country	Aim	Methodology	Sample	Types and scope	Input and output variables	Findings		Conclusions and limitations
							Efficiency level	Significant drivers and co-efficient and *p-value*	
									number of facilities included in the study was on the availability of data at the facility.
33.	Nundoochan (2020)<br><br>Mauritius	To assess the trend of technical efficiency in public regional hospitals in Mauritius	Stochastic frontier analysis (SFA) involving three functions: Cobb-Douglas; Translog; and Multi-output.	All five regional hospitals	Technical efficiency among regional hospitals	Input: Capital (the number of active beds), and labour (the sum total of all employees)<br><br>Output: Inpatients admitted and outpatients at the level of	Mean technical efficiency scores, using the Cobb Douglas, Translog and Multi-output functions for all the regional hospitals, were estimated at 83%, 84% and 89%, respectively.		Conclusion: The technical efficiency scores of the public regional hospitals were encouraging. However, further improvements on the efficiency of the regional hospitals could result in the freeing up of funds to finance other critical health programmes such

No.	Author, year<br><br>Country	Aim	Methodology	Sample	Types and scope	Input and output variables	Findings		Conclusions and limitations
							Efficiency level	Significant drivers and co-efficient and *p-value*	
						Accident, Emergency and uncategorised outpatient departments.			as combating vaccine preventable diseases and improving outbreak preparedness.<br><br>Limitation: The modeling was performed based on the assumption that all the patients that received healthcare at the facilities had similar profiles. Also, the modeling did not take into account potential

No.	Author, year<br>Country	Aim	Methodology	Sample	Types and scope	Input and output variables	Findings		Conclusions and limitations
							Efficiency level	Significant drivers and co-efficient and *p-value*	
									variations in the quality of care rendered across the hospitals. In addition, hospital beds were the only input variable used to represent capital resources.
34.	Obure et al., (2016)<br><br>Kenya and Swaziland	To determine technical efficiency of sexual and reproductive health services, and the effects of integration measures on	Data envelopment analysis	40 health facilities	Technical efficiency and its drivers in Kenya and Swaziland during 2008/09 and 2010/11	Input: labour (clinical staff and non-clinical staff), and expenditure on sexual and reproductive health services	The pooled mean bias corrected technical efficiency scores ranged from 22% to 65%. The bias corrected mean efficiency scores was 45% (standard DEA model with no quality measure), 65% (model with quality as an output), 49% (model with quality as input)	The number of additional HIV services in the maternal and child health unit (r=0.083, p<0.1), public ownership (r=0.353, p<0.05), and	Conclusion: There were low efficiency scores, and mixed effects of the measures of integration on the efficiency scores. This seems to suggest that integration of

No.	Author, year<br><br>Country	Aim	Methodology	Sample	Types and scope	Input and output variables	Findings		Conclusions and limitations
							Efficiency level	Significant drivers and co-efficient and *p-value*	
		technical efficiency				Outputs: Number of patients recorded receiving family planning, cervical cancer screening, postnatal care, HIV counselling and testing, treatment of sexually transmitted infections, and	and 22% (model with structural quality measure as input and process quality measure as output). In addition, the pooled mean uncorrected DEA bias corrected technical efficiency ranged from 60% to 84%. The bias corrected mean efficiency scores was 75% (standard DEA model with no quality measure), 84% (model with quality as an output), 79% (model with quality as input) and 60% (model with structural quality measure as input and process quality measure as output)	facility type (r=0.091, p<0.05), have a positive and significant effect on technical efficiency. However, number of additional HIV and STI services provided in the same clinical room (r=-0.096, p<0.05), proportion of	vertical services may not bring about a substantial increment in efficiency.<br><br>Limitation: The results of this study may not be generalised to other settings in sub-Saharan Africa.

No.	Author, year<br>Country	Aim	Methodology	Sample	Types and scope	Input and output variables	Findings		Conclusions and limitations
							Efficiency level	Significant drivers and co-efficient and *p-value*	
						HIV treatment and care services.		clinical staff to overall staff (r=-0.431, p<0.05), proportion of HIV services provided (r=-0.103, p<0.05), and rural location had a negative and significant effect on technical efficiency	
35.	See and Yen (2018)	To assess the performance of health systems in	Double bootstrap data envelopment analysis	22 African countries	Technical efficiency among 121 countries	Input: Health expenditure, labour, hospital	The technical efficiency scores were reported as bias-corrected efficiency scores. The closer the bias-corrected	Regression analysis of inefficiency against	Conclusion: Initiatives that improve happiness of people can

No.	Author, year<br><br>Country	Aim	Methodology	Sample	Types and scope	Input and output variables	Findings		Conclusions and limitations
							Efficiency level	Significant drivers and co-efficient and *p-value*	
	Multiple countries	countries across the globe, and the influence of happiness on the performance of the health systems			worldwide, consisting of 22 African countries	beds and education.<br><br>Output: Healthy life expectancy and inverse mortality index.	efficiency score of a country is to 1, the more efficient the health systems of the country are. The scores of upper-middle income African countries included in the study: Libya (1.0677); Namibia (1.1462); and Botswana (1.1775). The scores of the lower-middle income African countries included: Tunisia (1.0161); Morocco (1.0225); Ghana (1.0854); Sudan (1.1003); Kenya (1.1039); Zambia (1.1438); Cameroon (1.1830). The efficiency scores of low-income countries were dominated by	variables such as happiness ($r=-0.2478$, $p<0.01$), population density ($r=-0.0187$, $p<0.01$), and healthcare share of GDP ($r= -0.0399$, $p<0.05$) were all significant.	ultimately enhance the efficiency of health systems.<br><br>Limitation: There were limitations with the data that was used. Firstly, data was not available for some countries on some of the variables. Secondly, the only data from 2014 was used.

No.	Author, year<br><br>Country	Aim	Methodology	Sample	Types and scope	Input and output variables	Findings		Conclusions and limitations
							Efficiency level	Significant drivers and co-efficient and *p-value*	
							African countries. Out of the 16 low-income countries included in the study, there was 15 African countries. The efficiency scores of the low-income African countries are as lows: Rwanda (1.0169); Malawi (1.0193): Ethiopia (1.0331); Senegal (1.0332); Burkina Faso (1.0337); Central African Republic (1.0339); Mali (1.0342); Madagascar (1.0342); Togo (1.0342); Liberia (1.0344); Benin (1.0972); Tanzania (1.1053); Uganda (1.1064); Zimbabwe (1.1376); and Mozambique (1.1450).		

No.	Author, year<br><br>Country	Aim	Methodology	Sample	Types and scope	Input and output variables	Findings		Conclusions and limitations
							Efficiency level	Significant drivers and co-efficient and *p-value*	
36.	Yitbarek et al., (2019)<br><br>Ethiopia	To investigate the technical efficiency of primary health systems that provide neonatal health services in Southwest Ethiopia.	Data envelopment analysis	23 health centres and 68 health posts	Technical efficiency of neonatal health services in Southwest Ethiopia	Input: For the health centres included: non-salary recurrent expenses (expenses for vaccine, drug and supplies); administrative staff; clinical and midwife nurses; laboratory technicians and technologists; pharmacy technicians and pharmacists; and health	Regarding the health posts, the mean technical efficiency score was 42%. Only 11.76% of the health posts were operating at 100% technical efficiency. Also, regarding the health posts, 5.88% were 76% to 99% efficient, 17.65% were 51% to 75% efficient, 22.06% were 26% to 50% efficient, majority (42.65%) were less than 25%.<br><br>Regarding the technical efficiency of health centres the mean efficiency score	Regarding the health centres, the number of years of experience of the leader of the facility ($r=0.013$, $p<0.01$), and the catchment population of the facility ($r=0.0001$, $p<0.05$) were positively associated with technical efficiency. Regarding the	Conclusion: Technical efficiency in the primary healthcare system is below satisfaction. Moreover, the efficiency is much worse in health posts than health centres. It may be possible to increase the efficiency of health systems by giving training to heads of the facilities towards

No.	Author, year<br><br>Country	Aim	Methodology	Sample	Types and scope	Input and output variables	Findings		Conclusions and limitations
							Efficiency level	Significant drivers and co-efficient and *p-value*	
						officers. The input variables for the health posts included: non-salary recurrent expenses (vaccine, medicine and supplies); and health extension workers.<br><br>Output: The health centre outputs	was 75%. Out of the health centres, 34.8% were 100% efficient, 21.7% were 76% to 99% efficient, 21.7% were 51% to 75% efficient, 17.4% were 26% to 50% efficient, and 4.3% were less than 25% efficient.	health posts, neonates within the catchment population was positively associated with efficiency (r=0.0006, p<0.05). To the contrary, waiting time in the health post was negatively associated with efficiency (r=-0.01, p<0.05).	reducing the waiting time.<br><br>Limitation: Social and behavioural factors which could affect output were not investigated. In addition, a proportion of the entire healthcare expenditure was taken in order to estimate the input for neonatal health services. So, the

No.	Author, year<br><br>Country	Aim	Methodology	Sample	Types and scope	Input and output variables	Findings		Conclusions and limitations
							Efficiency level	Significant drivers and co-efficient and *p-value*	
						included: the number of neonatal outpatients; neonatal referral and those who received service at maternal; and child health units. The outputs for the health post include: neonatal outpatients; neonatal referrals to			input data may not be accurate.

No.	Author, year<br><br>Country	Aim	Methodology	Sample	Types and scope	Input and output variables	Findings		Conclusions and limitations
							Efficiency level	Significant drivers and co-efficient and *p-value*	
						health centres; and home-to-home service.			
37.	Yitbarek et al., (2019)<br><br>Ethiopia	To assess technical efficiency of maternal and reproductive services and its determinant among public hospitals in the Oromia Regional	Data envelopment analysis	14 hospitals	Technical efficiency on maternal and reproductive health services among hospitals in the Oromia Regional State.	Input: Total expenditure for maternal and reproductive health service, including capital, human resources, non-salary expenditures (drugs, vaccines, and medical supplies), and	Twelve, representing 85.71% of the hospitals were 100% technically efficient, and the mean technical efficiency score of all the hospitals was 85%.	The level of the health facility ($r=1.17$, $p<0.05$), number of years of duration of the operation of the hospital ($r=0.02$, $p<0.05$), and the size of the catchment	Conclusion: A huge majority of the hospitals were technically efficient. There is the opportunity to make further judicious use of resources by expanding health services in older secondary hospitals with larger

No.	Author, year<br>Country	Aim	Methodology	Sample	Types and scope	Input and output variables	Findings		Conclusions and limitations
							Efficiency level	Significant drivers and co-efficient and *p-value*	
		State, Ethiopia				salary for the staff.<br><br>Output: Numbers of antenatal care visits, delivery, postnatal care, family planning, abortion and post- abortion services delivered in the hospitals.		population of the hospital ($r=5.58E–07$, $p<0.05$) were positively associated with technical efficiency. However, the average waiting time at the hospitals was negatively associated with technical efficiency ($r=-0.03$, $p<0.05$).	catchment population.<br><br>Limitations: Certain socio-cultural, and individual factors which have the potential to influence output in health systems were not considered. Also, the inputs used for the study were general hospital inputs. In order to

No.	Author, year<br><br>Country	Aim	Methodology	Sample	Types and scope	Input and output variables	Findings		Conclusions and limitations
							Efficiency level	Significant drivers and co-efficient and *p-value*	
									compute the technical efficiency of maternal and reproductive services, an estimation was made on the proportion of the general inputs that were used for maternal and reproductive health services. As such, it was possible that the input was either over or underestimated.

No.	Author, year<br><br>Country	Aim	Methodology	Sample	Types and scope	Input and output variables	Findings		Conclusions and limitations
							Efficiency level	Significant drivers and co-efficient and *p-value*	
38.	Zarulli et al., (2021)<br><br>Multiple countries	To determine the efficiency of health systems and estimate the contribution of healthcare spending to life expectancy	Data envelopment analysis	140 countries worldwide, consisting of 43 countries in Africa	Efficiency	Variables were not classified into input and output variables. Education Index, infants lacking immunization against diphtheria, pertussis and tetanus and measles vaccines, current health expenditure as a percentage of GDP, share of	The efficiency scores of African countries included in the study were: Angola (1.22); Benin (1.14); Botswana (1.15); Burkina Faso (1.2); Burundi (1.03); Cabo Verde (1.04); Cameroon (1.25); Chad (1.34); Central African Republic (1.15); Comoros (1.15); Congo (1.16); DR Congo (1); Egypt (1.07); Swaziland (1.37); Ethiopia (1.03); Gabon (1.18); Gambia (1.1); Ghana (1.16); Guinea (1.2); Guinea-Bissau (1.26); Kenya (1.11); Lesotho (1.4); Liberia (1.15); Madagascar (1.09); Malawi		Conclusion: Decreasing unemployment rate and income disparities in countries with levels of education would result in improving efficiency without increasing health expenditure.<br><br>Limitation: Limited number of variables were used due to limited availability

No.	Author, year<br>Country	Aim	Methodology	Sample	Types and scope	Input and output variables	Findings		Conclusions and limitations
							Efficiency level	Significant drivers and co-efficient and *p-value*	
						population using at least basic sanitation services, share of unemployed in the labour force, income inequality measured by the Gini coefficient, and the ratio of 65 or older population to those of working age.	(1.15); Mali (1.21); Mauritania (1.33); and Mauritius (1.07). The efficiency scores of the other countries were: Mozambique (1.13); Namibia (1.26); Niger (1.08); Nigeria (1.38); Rwanda (1.07); Sao Tome (1.05); Rwanda (1.07); Senegal (1.08); Sierra Leone (1.38); South Africa (1.25); Sudan (1.14); Tanzania (1.13); Togo (1.20); Tunisia (1.03); Uganda (1.16); Zambia (1.17); and Zimbabwe (1.22).		of data. Also, the variables which were used in the study were population level variables, so it may not be appropriate to draw conclusions at the individual level.
39.	Zeng et al. (2014)	To assess the technical efficiency of	Data envelopment analysis	26 health centres	Technical efficiency among	Input: Number of HIV/AIDS staffs, and	The overall efficiency of the health centres from 2006 to 2007 was 78%. In 2006, the		Conclusion: The overall efficiency

No.	Author, year<br>Country	Aim	Methodology	Sample	Types and scope	Input and output variables	Findings		Conclusions and limitations
							Efficiency level	Significant drivers and co-efficient and *p-value*	
	Rwanda	rural health centres in delivery HIV/AIDS related services			health centres in rural parts of Rwanda in delivering HIV/AIDS services	expenditure on HIV/AIDS medical logistics.<br><br>Output: Number of clients receiving voluntary counselling and testing, number of participants in prevention of mother-to-child	mean efficiency score among all the health centres was 82%. In the same year, 11 out of the 26 health centres recorded 100% efficiency. In 2007, the mean efficiency score among all the health centres was 73.9%. in the same year, 8 out of the 26 health centres were 100% efficient. In 2006, only one of the health centres had an efficiency score below 50%, but in 2007, three of the health centres had efficiency scores below 50%.		leaves much to be desired.<br><br>Limitation: It would have been insightful to establish a relationship between performance-based financing (which was also assessed in the study) and health efficiency, but the relatively small sample size used in the study

No.	Author, year<br><br>Country	Aim	Methodology	Sample	Types and scope	Input and output variables	Findings		Conclusions and limitations
							Efficiency level	Significant drivers and co-efficient and *p-value*	
						transmission, and number of AIDS patients receiving antiretroviral therapy.			did not make this feasible. The study took a proportion of the entire healthcare expenditure to estimate HIV/AIDS expenditure, so it was possible that either this aspect of the input data was either understated or overstated. In the event that it was overstated, then the efficiency was higher, but in the event that it was understated, then

No.	Author, year<br><br>Country	Aim	Methodology	Sample	Types and scope	Input and output variables	Findings		Conclusions and limitations
							Efficiency level	Significant drivers and co-efficient and *p-value*	
									the efficiency was lesser.

Table S3: Inputs and outputs variables and drivers of (in)efficiency

Health system level efficiency studies

Input variable	Studies	Frequency
Total recurrent expenditures, Out-of-pocket expenditure; current health expenditure	Sun et al., (2017); Nassar et al., (2020); Obure et al., (2016); Jordi et al., (2020); Top et al., (2019); Ibrahim et al., (2018); Sinimole (2012); Zarulli et al., (2021); Grigoli and Kapsoli (2018); Kim and Kang (2014)	10
Workforce/ labour	See and Yen (2018); Obure et al., (2016); Top et al., (2019); Di Giorgio et al., (2016); Grigoli and Kapsoli (2018); Kim and Kang (2014);	6
Bed capacity	See and Yen (2018); Top et al., (2019); Di Giorgio et al., (2016);	3
Unemployment rate	Top et al., (2019); Zarulli et al., (2021)	2
Immunization rate	Ibrahim et al., (2018); Zarulli et al., (2021)	2
Gini coefficient	Top et al., (2019); Zarulli et al., (2021)	2
Education index	Nassar et al., (2020); Zarulli et al., (2021)	2
Age group	Sinimole (2012); Zarulli et al., (2021)	2
Food production index	Nassar et al., (2020);	1
Inflation rate	Nassar et al., (2020)	1
Purchasing power parity	Nassar et al., (2020)	1
urbanization	Nassar et al., (2020)	1
External resources	Sinimole (2012)	1
Use of sanitation services	Zarulli et al., (2021)	1

Health facility level studies

Input variable	Studies	Frequency
Workforce	Kinyanjui et al., (2015); Anselmi et al., (2018); Kirigia and Asbu (2013); Marschall and Flessa (2011); Aduda et al., (2015); Mujasi et al., (2016); Novignon and Nonvignon (2017); Bobo et al., (2018); Kirigia et al., (2011), Achoki et al. (2017); Jehu-Appiah et al (2014); Nundoochan (2020); Yitbarek et al., (2019); Zeng et al. (2014); Babalola, and Moodley (2020); Ali et al., (2017); Atake (2019); Ichoku et al., (2011); Kinfu (2013); Ngobeni et al., (2020); Amponsah and Amanfo (2016; Alhassan et al., (2015); Jarue et al., (2015)	23
Bed capacity	Kinyanjui et al., (2015); Kirigia and Asbu (2013); Aduda etl al., (2015); Amare et al., (2020); Mujasi et al., (2016); Novignon and Nonvignon (2017); Jehu-Appiah et al (2014); Nundoochan (2020); Babalola, and Moodley (2020); Ali et al., (2017); Atake (2019); Ichoku et al., (2011); Kinfu (2013); Alhassan et al., (2015); Jarjue et al., (2015)	15
Total expenditure/recurrent of the health facility	Yitbarek et al., (2019); Anselmi et al., (2018); Marschall and Flessa (2011); Amare et al., (2020); Novignon and Nonvignon (2017); Ichoku et al., (2014); Jehu-Appiah et al (2014); Yitbarek et al., (2019); See and Yen (2018); Ichoku et al., (2011); Kinfu (2013); Ngobeni et al., (2020); Achoki et al. (2017) ;Zeng et al. (2014);	14
Capital cost	Ichoku et al., (2014)	1
Number of admissions	Ichoku et al., (2014)	1
Number of Xrays	Ichoku et al., (2014)	1
Equipment index	Anselmi et al., (2018)	1
Area	Marschall and Flessa (2011)	1
Depreciation	Marschall and Flessa (2011)	1
Vaccine	Marschall and Flessa (2011)	1
Time spent on service	Aduda etl al., (2015)	1
Medicines	Ali et al., (2017)	1

Insurance coverage	Kinfu (2013)	1
Access to water	Kinfu (2013)	1

System wide studies Outputs variables

Output variable	Reference	Frequency
Life expectancy/ Health adjusted life years	Sun et al., (2015); See and Yen (2018); Nassar et al., (2020); Top et al., (2019); Ibrahim et al., (2018); Sinimole (2012); Grigoli and Kapsoli (2017); Kim and Kang (2014)	8
Mortality rate	Sun et al., (2015); See and Yen (2018); Top et al., (2019); Ibrahim et al., (2018); Sinimole (2012); Grigoli and Kapsoli (2017); Kim and Kang (2014)	7
Outpatient visits	Obure et al., (2016); Jordi et al., (2020); Di Giorgio et al., (2016);	3
Inpatient admissions	Jordi et al., (2020); Di Giorgio et al., (2016);	2
Infection rate	Ibrahim et al., (2018); Sinimole (2012)	2
Immunization rates	Sinimole (2012); Grigoli and Kapsoli (2017);	2
Antenatal care coverage	Sinimole (2012);	1
ART visits	Di Giorgio et al., (2016);	1
Number of deliveries	Di Giorgio et al., (2016);	1
Current health expenditure	Jordi et al., (2020)	1
Access to healthcare	Jordi et al., (2020)	1

Skilled attendance at labour	Sinimole (2012)	1
Treatment success rates	Grigoli and Kapsoli (2017)	1

Health facility level output variables

Output variable	Reference	Frequency
Outpatient visits	Kinyanjui et al., (2015); Anselmi et al., (2018); Marschall and Flessa (2011); Mujasi et al., (2016); Novignon and Nonvignon (2017); Bobo et al., (2018); Kirigia et al., (2011); Jehu-Appiah et al (2014); Yitbarek et al., (2019); Zeng et al. (2014); Babalola, and Moodley (2020); Ali et al., (2017); Ichoku et al., (2011); Jarue et al., (2015)	14
Mortality rate	Achoki et al. (2017); Kinfu (2013); Ngobeni et al., (2020); Amponsah and Amanfo (2016);	4
Inpatients admitted	Kinyanjui et al., (2015); Kirigia and Asbu (2013); Mujasi et al., (2016); Jehu-Appiah et al (2014); Nundoochan (2020); Yitbarek et al., (2019); Babalola, and Moodley (2020); Ali et al., (2017); Atake (2019); Ichoku et al., (2011); Jarue et al., (2015)	11
Number of deliveries	Marschall and Flessa (2011); Mujasi et al., (2016); Bobo et al., (2018); Jehu-Appiah et al (2014); Atake (2019); Kinfu (2013); Alhassan et al., (2015)	37
Laboratory tests	Jehu-Appiah et al (2014); Ichoku et al., (2011)	2
Surgical operations	Babalola, and Moodley (2020); Atake (2019)	2
Number of X-rays done	Babalola, and Moodley (2020); Ichoku et al., (2011)	2
Immunization rates	Marschall and Flessa (2011); Bobo et al., (2018); Kirigia et al., (2011); Alhassan et al., (2015)	4

Skilled attendance at birth	Amare et al., (2020)	1
Antenatal care coverage	Yitbarek et al., (2019); Amare et al., (2020); Bobo et al., (2018);	4
ART visits	Aduda etl al., (2015); Alhassan et al., (2015)	2
Treatment success rates	Kirigia and Asbu (2013).	1
Postnatal coverage	Yitbarek et al., (2019); Amare et al., (2020)	2
Number of reproductive and family planning visits	Yitbarek et al., (2019); Bobo et al., (2018); Kirigia et al., (2011)	3
Quality of service	Aduda etl al., (2015)	1
Postnatal coverage	Alhassan et al., (2015)	1
Number of reproductive and family planning visits	Alhassan et al., (2015)	1
Competition with other health facilities	Atake (2019)	1
